# Risk of Developing Non-Cancerous Central Nervous System Diseases Due to Ionizing Radiation Exposure during Adulthood: Systematic Review and Meta-Analyses

**DOI:** 10.3390/brainsci12080984

**Published:** 2022-07-26

**Authors:** Julie Lopes, Klervi Leuraud, Dmitry Klokov, Christelle Durand, Marie-Odile Bernier, Clémence Baudin

**Affiliations:** 1 Laboratory of Epidemiology (LEPID), Institute for Radiobiological Protection and Nuclear Safety (IRSN), 92262 Fontenay-aux-Roses, France; klervi.leuraud@irsn.fr (K.L.); marie-odile.bernier@irsn.fr (M.-O.B.); clemence.baudin@irsn.fr (C.B.); 2 Experimental Radiotoxicology and Radiobiology Laboratory (LRTOX), Institute for Radiobiological Protection and Nuclear Safety (IRSN), 92262 Fontenay-aux-Roses, France; dmitry.klokov@irsn.fr (D.K.); christelle.durand@irsn.fr (C.D.)

**Keywords:** systematic review, meta-analyses, ionizing radiation, central nervous system, mental health, cognitive disorders, cerebrovascular diseases, mental and behavioral disorders, epidemiology

## Abstract

Background: High-dose ionizing radiation (IR) (>0.5 Gy) is an established risk factor for cognitive impairments, but this cannot be concluded for low-to-moderate IR exposure (<0.5 Gy) in adulthood as study results are inconsistent. The objectives are to summarize relevant epidemiological studies of low-to-moderate IR exposure in adulthood and to assess the risk of non-cancerous CNS diseases. Methods: A systematic literature search of four electronic databases was performed to retrieve relevant epidemiological studies published from 2000 to 2022. Pooled standardized mortality ratios, relative risks, and excess relative risks (ERR) were estimated with a random effect model. Results: Forty-five publications were included in the systematic review, including thirty-three in the quantitative meta-analysis. The following sources of IR-exposure were considered: atomic bomb, occupational, environmental, and medical exposure. Increased dose-risk relationships were found for cerebrovascular diseases incidence and mortality (ERR_pooled_ per 100 mGy = 0.04; 95% CI: 0.03–0.05; ERR_pooled_ at 100 mGy = 0.01; 95% CI: −0.00–0.02, respectively) and for Parkinson’s disease (ERR_pooled_ at 100 mGy = 0.11; 95% CI: 0.06–0.16); Conclusions: Our findings suggest that adult low-to-moderate IR exposure may have effects on non-cancerous CNS diseases. Further research addressing inherent variation issues is encouraged.

## 1. Introduction

Recent decades have seen an increase in the exposure of the overall population to ionizing radiation (IR), especially due to the widespread use of medical imaging procedures in economically developed countries [1]. Nowadays, the average annual effective IR-dose is estimated to be around 3.0 millisievert (mSv) per person, including 20% from medical exposure [2]. The latter tends to have increased from year to year due to the use of new technological imaging from around 0.3 mSv in 1993 to 0.6 mSv in 2021 [2].

The adverse health effects following exposure to IR have been the subject of a large amount of scientific research, mainly focusing on carcinogenic risks [3]. However, several epidemiological studies have highlighted the non-cancerous detrimental impact of high or moderate IR doses on the central nervous system (CNS) [4,5,6], and radiation-induced cognitive injury is becoming an increasingly important research subject [7,8]. Non-cancerous CNS disorders are a large and complex group of diseases, including mental and behavioral disorders, diseases of the CNS, and trauma, and they have multifactorial origins. The Institute for Health Metrics and Evaluation estimates that in 2019, 970 million people suffered from a mental disorder and 2.7 billion people had neurological disorders [9]. In addition to the negative consequences of these pathologies on individual well-being, these disorders cost several trillion U.S. dollars each year in the global economy.

Neurodevelopmental effects of high doses of IR exposure during childhood are well established, but the extent to which these effects exist in the low-to-moderate-dose range is unclear [10]. For this reason, most studies have investigated the impact of low-to-moderate IR doses (<0.5 Gy) on cognitive functions, when exposure occurred in utero or during childhood, but findings remain inconsistent, particularly in view of the large number of assessment tools and scales available to assess neurocognitive disorders [10]. In contrast, few studies have considered these outcomes when exposure occurred in adulthood [11]. It has been suggested that low-dose IR exposure during adulthood could enhance the incidence of certain neurodegenerative diseases, such as Parkinson’s disease [12] and cerebrovascular diseases [13].

A relevant point is that experimental studies in rodents can help detect cognitive effects that may occur after IR exposure (doses ≤ 1 Gy) in adulthood, although the translation of data from animal experiments to humans is challenging. Some forms of memory and social interaction can be impaired after acute [14,15,16,17,18] or chronic [19] exposures in adult rodents. Moreover, internal acute or chronic exposure to uranium, using different routes of exposure, can also have a deleterious impact on certain forms of memory [20,21,22,23], and an effect of the enriched form of uranium is more deleterious compared with depleted uranium [22]. Furthermore, few hours after low-dose (<0.1 Gy) brain IR exposure, the downregulation of molecular neural pathways associated with cognitive decline and Alzheimer’s disease has been observed in adult mice [24]. Altogether, these experimental data suggest that low-to-moderate doses of IR in adulthood can impact neurocognitive functions in rodents under certain conditions and highlight the need to investigate further the potential effects of this type of exposure, particularly in humans.

Thus, the objectives of the present systematic review are to (1) identify pertinent studies, synthesize their results, and draw evidence-based conclusions from epidemiological studies carried out on the risk of non-cancerous CNS diseases (e.g., cerebrovascular, neurological, and psychiatric diseases, such as neurodegenerative, mental, and behavioral disorders), in adults exposed to low-to-moderate doses of IR (<0.5 Gy), and (2) to provide a quantitative summary of the overall risk estimate using meta-analysis.

## 2. Methods

This literature review and synthesis were guided by the Preferred Reporting Items for Systematic Review and Meta-Analyses (PRISMA) guidelines (Table S1), and the protocol was recorded in the PROSPERO database (registration number: CRD42021283245).

### 2.1. Data Source and Search

An online literature search was conducted in May 2022 in PubMed, Scopus, Web of Science, and Google Scholar databases. A first query included a combination of outcome, exposure, and population keywords: (neuro * OR nervous OR cognit * OR Parkinson OR Alzheimer OR brain OR cerebro * OR dementia OR schizophrenia OR cerebrovascular) AND (ionizing radiation OR medical radiation OR cosmic radiation OR nuclear OR radon OR background radiation) AND (patient * OR human OR worker OR cohort OR epidemiolog *). Subsequently, additional queries were used to complete this previous one in order to identify studies without the keywords in the title or abstracts: cosmic radiation AND mortality OR incidence; (nuclear worker OR nuclear facility OR nuclear industry) AND mortality OR incidence; ionizing radiation AND mortality OR incidence. Subsequently, additional articles were searched from the references cited by relevant publications and international reports. Duplicates from the different databases were removed.

For the selection process, we proceeded as follows: (1) the articles obtained through the queries were screened on title; (2) the abstracts of the selected articles were read, and a further selection was performed; (3) the articles were selected on the full-text screening. The selection was carried out by two independent investigators (J.L. and C.B.), whereas a third investigator (M.-O.B.) made a decision in the case of disagreement.

### 2.2. Inclusion and Exclusion Criteria

Eligible studies were cohort, case–control, and cross-sectional studies, published in English from January 2000 to May 2022. The publication period criterion allows for the inclusion of studies whose radiation exposure reflects the improvement in radiation protection regulations and the decrease in doses received by medical professionals [25]. Furthermore, older good-quality studies are regularly updated and would be found as their last updated publication. All the studies focused on external (e.g., gamma rays, X-rays, cosmic rays) or internal (e.g., uranium, plutonium, radon) exposures to low-to-moderate doses of IR (mean: 0.5 Gy) occurring during adulthood or adolescence (at least 16 years old) as companies involved in some of the studies included in this work allowed for work at 16 or older. Incidence and/or mortality of three categories of non-cancerous CNS diseases, identified with the International Classification of Diseases (ICD), Revision 9/10 [26], were studied in this work: diseases of the nervous system (ICD-9: 320–389/ICD-10: G00–G99), cerebrovascular diseases (ICD-9: 430–438/ICD-10: I60–I69), and mental and behavioral disorders (ICD-9: 290–319/ICD-10: F00–F99). Studies that did not report an ICD classification but referred to “diseases of the nervous system”, “cerebrovascular diseases”, and “mental and behavioral disorders” to describe their outcomes of interest were also investigated and classified in the groups mentioned above, respectively. The definitions of the ICD codes are provided in the Supplementary Materials (Table S2).

Conference abstracts, reports, meta-analyses, letters, and ecological studies were excluded, as well as studies where exposure information was based on self-reports or questionnaires about IR exposure (e.g., “How many dental X-rays have you been exposed to in your lifetime?”). However, the references of these excluded studies were checked to retrieve potential studies that met the inclusion criteria of the present review. In the case of publications on overlapping populations or study updates, only data from the most complete study were considered.

### 2.3. Quality Assessment of Individual Studies

The Newcastle Ottawa Scale (NOS) was used for quality assessment of the epidemiological studies included in this review [27], which is usually used in systematic review process. This evaluation is based on eight items, which are categorized into three groups: selection of study groups, comparability of groups, and ascertainment of exposure or outcome of interest, for case–control or cohort studies. Stars are attributed to each item depending on the quality, and a score (0 to 9) is obtained by adding the stars of each item. A study with an average NOS score of at least 6 stars out of 9 is considered as having good quality.

### 2.4. Statistical Analysis

Estimates of measures of risk such as relative risk (RR), hazard ratio (HR), odds ratio (OR), or standardized mortality ratio (SMR) and measures of risk by unit of dose such as excess relative risk (ERR) were extracted from each study when available. Meta-analyses for each outcome were performed if at least a sufficient number (at least 3) of studies were available.

We calculated pooled SMR (SMR_pooled_) and pooled RR estimates (RR_pooled_) and their 95% confidence interval (CI) using the DerSimonian and Laird random-effects model [28] to account for within- and between-study heterogeneities.

An alternative DerSimonian and Lair-based model proposed by Richardson et al. (2020) was used to estimate the pooled effect of ERRs [29]. This method for meta-analysis of published results from linear relative risk models uses a parametric transformation of published results to improve on the normal approximation used to assess confidence intervals. This approach provides less biased summary estimates with better confidence-interval coverage than the summary obtained using the more classical approach to meta-analysis.

Heterogeneity across studies was tested using Cochran’s Q test at *p* < 0.1 and quantified using I^2^ statistics. The latter reflects the proportion of total variance estimated to be attributable to between-study heterogeneity. Heterogeneity was considered as null, low, moderate, and high for I^2^ values < 25, 25–50, 50–75, and >75%, respectively. In the case of heterogeneity, sensitivity analyses in which the pooled result was calculated by excluding each study and each group of workers in turn were performed. Sensitivity analyses were also performed, removing studies that did not mention ICD coding in the outcome definition. Publication and selection biases were assessed and tested using the Egger test. Statistical significance was defined by *p* < 0.05.

Statistical analyses were conducted with the R 3.6.3 software (R Foundation for Statistical Computing, Vienna, Austria) using the metafor and Metaan packages.

## 3. Results

**Figure 1:**
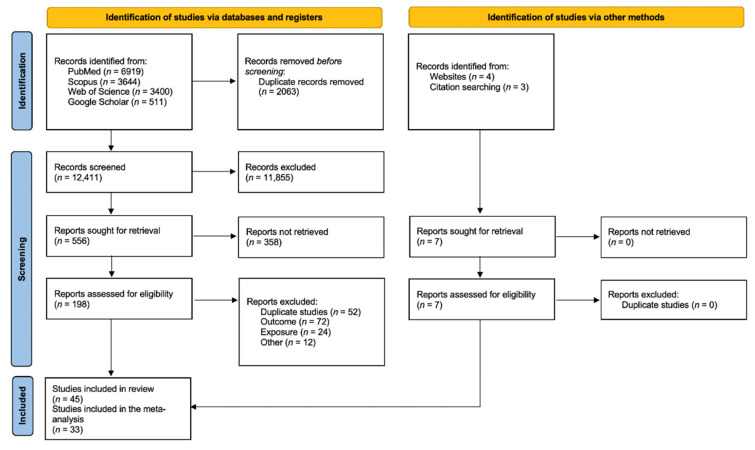
Preferred Reporting Items for Systematic Reviews and Meta-Analyses 2020 flow diagram for new systematic reviews which included searches of databases, registers, and other sources.

From the 14,474 articles retrieved, 2063 were excluded as being duplicates and 12,411 were screened based on the title, which led to the review of 556 abstracts. Finally, 198 articles were read in full, of which 38 were selected. Briefly, full texts were excluded because of overlaps (58 studies), outcome not in the scope of the review (72 studies), study design not meeting inclusion criteria (24 studies), or for other reasons (6 studies, for a combination of several exclusion criteria). Seven additional articles were identified from bibliographic references of the retrieve articles, thus leading to forty-five articles finally included in the systematic review. Of those, 33 presented quantitative results that could be included in the meta-analyses (Figure 1). The characteristics and NOS score assessments of the 45 articles included in the present review are detailed in Table 1. There were forty cohort studies, one case–control study, and four cross-sectional studies. Most of the studies investigated non-cancerous CNS diseases in relation to occupationally exposed workers (forty-two studies), while others addressed environmental exposure (one study), evacuees from Chernobyl (one study), or patients exposed for medical purposes (one study).

### 3.1. Diseases of the Nervous System (ICD-10: G00–G99)

Key findings of the 21 studies focusing on diseases of the nervous system can be found in Table 2.

#### 3.1.1. Nuclear Workers and Uranium Miners

Out of the 21 studies that considered this outcome, 13 were on nuclear workers and uranium miners. The majority of them did not report any statistically significant results, whether the authors compared the mortality of workers to that of an external reference population (SMR) or they assessed dose–response relationships (ERR) [33,35,36,39,42,44,46].

In a cohort of 4977 U.S. mound workers potentially exposed to external or internal (polonium-210, plutonium isotopes, or tritium) radiation (mean dose from external radiation: 26.1 mSv; max: 939.1 mSv; mean lung dose from external and internal radiation combined: 100.1 mSv; max: 17.5 Sv; mean liver dose from external and internal radiation: 34.6 mSv; max: 2.3 Sv), the mortality due to diseases of the nervous system was not different from that of the general population, regardless of the radiation status of the workers or the type of radionuclides for those with intakes, but a positive trend was suggested as SMRs increased with increasing categories of occupational cumulative dose primarily due to photons (*p* = 0.03) [42]. In a cohort of 26,328 Los Alamos National Laboratory workers exposed to a combination of photons, neutrons, tritium, and plutonium (among which 17,053 workers were monitored for a combination of external and internal sources for plutonium; brain radiation absorbed dose: mean: 11.6 mGy; max: 760 mGy), Boice et al. (2021) reported among the whole cohort a non-significant SMR for nervous system diseases compared with national rates based on 815 deaths, but a borderline significant increase in mortality due to Parkinson’s disease was observed (SMR = 1.16; 95% CI: 1.00, 1.34; n_deaths_ = 193), and a positive dose–response relationship was suggested (ERR at 100 mGy = 0.16; 95% CI: −0.07, 0.40; n_deaths_ = 273) [33]. In a cohort of 22,377 Mayak workers exposed to chronic IR (mean cumulative dose from external gamma rays absorbed in the brain: 0.46 Gy (max: 8.01 Gy) for men and 0.36 Gy (max: 6.14 Gy) for women), a statistically significant linear association was found between the incidence of Parkinson’s disease and cumulative gamma dose after adjusting for sex and attained age (ERR per Gy = 1.02; 95% CI: 0.59, 1.63; n_diseases_ = 300) [12]. A significant decrease in mortality was reported among 53,698 U.S. nuclear power plant industry workers (mean cumulative dose: 25.7 mSv) compared with the U.S. general population (SMR= 0.50; 95% CI: 0.31, 0.77; n_deaths_ = 20), whereas a positive and statistically significant, but highly imprecise, dose–response relationship was observed (ERR per Sv= 46.8; 95% CI: 1.51, 242; n_death_ = 20) [49]. Later, Boice et al. (2021) also reported a significant decrease in mortality from diseases of the nervous system compared with national rates in 135,193 U.S. nuclear power workers (mean dose to the brain: 33.2 mGy; max: 0.83 Gy) (SMR = 0.82, 95% CI: 0.76, 0.89; n_deaths_ = 673), and the authors observed a positive non-significant dose–response relationship for Parkinson’s disease mortality (ERR at 100 mGy = 0.24; 95% CI: −0.02, 0.50; n_deaths_ = 140) [32].

In studies on uranium miner, miller, or processing workers, inconsistent results were observed, as a significantly increased mortality from diseases of the nervous system was observed in 2,930 uranium miners and millers of Grants (New Mexico) potentially exposed to radon, radon decay products, uranium dust and mill products (exposure assessment: NA) compared with the general U.S. population (SMR= 1.60; 95% CI: 1.01, 2.39; n_deaths_ = 23) [47], while a significantly decreased mortality was found in 16,236 male Eldorado uranium workers exposed to gamma rays (dose: 52.2 mSv) or/and radon decay products (100.2 WLM) compared with the general Canadian male population (SMR = 0.66; 95% CI: 0.51, 0.85; n_deaths_ = 61) [45] and in 35,204 male underground uranium miners of the WISMUT AG exposed to radon (mean: 364 WLM) or/and crystalline silica (mean: 7.6 mg/m^3^ years) compared to the general male population in Eastern Germany (SMR = 0.73; 95% CI: 0.62, 0.85; n_deaths_ = 163) [34].

#### 3.1.2. Nuclear Weapons Test Participants

Concerning nuclear weapons test participants, a significant decrease in mortality from diseases of the nervous system was observed among 114,270 male U.S. military participants in atmospheric tests in Nevada and the Pacific from 1945 to 1962 (mean NuTRIS film badge gamma radiation dose: 6 mSv; max: 908 mSv) compared to the general male population of the U.S. (SMR = 0.84; 95% CI: 0.81, 0.88; n_deaths_ = 1871) [53]. The 10,983 male Australian participants in the British nuclear tests conducted in Australia between 1952 and 1957 (mean radiation exposure: 2.8 mSv; max > 50 mSv) showed similar mortality to that of the general male population for diseases of the nervous system (SMR = 1.02; 95% CI: 0.78, 1.32; n_deaths_ = 59), but showed a non-significantly higher mortality for motor neuron disease (SMR = 1.24; 95% CI: 0.71, 2.02; n_deaths_ = 16) [54].

#### 3.1.3. Chernobyl Cleanup Workers

Rahu et al. (2014) reported an increased risk of diseases of the nervous system in a cohort of 3680 Estonian Chernobyl cleanup workers recruited between 1986 and 1991 to the Chernobyl area by the Soviet authorities for decontamination, building, and other related activities compared to a population-based cohort of 7631 unexposed Estonian men (RR = 1.13, 95% CI: 1.06, 1.21) [70]. However, the accuracy of the diagnosis and the representativeness of the unexposed cohort are an issue of this study.

#### 3.1.4. Medical Workers

In a cohort of 109,019 U.S. medical and associated radiation workers exposed to X- and gamma rays (mean cumulative absorbed dose to the brain: 18.9 mGy; max: 1.08 Gy), monitored between 1965 and 1994 and followed-up until 2016, a significant decrease in mortality from diseases of the nervous system (SMR = 0.72; 95% CI: 0.65, 0.78; n_deaths_ = 471) was found, but not for mortality from Parkinson’s disease (SMR= 0.82; 95% CI: 0.66, 1.02; n_deaths_ = 84) when compared with the general population, and a non-significant positive dose response for Parkinson’s disease was found (ERR at 100 mGy = 0.17; 95% CI: −0.20, 0.54; n_deaths_ = 87) [58]. Furthermore, no significant increase in mortality from diseases of the nervous system nor for Alzheimer’s disease was found among 34,912 U.S. male radiologists (exposure assessment: NA) when compared to 47,497 male psychiatrists or to the general population (including both men and women) [62], and no increased risk of mortality from diseases of the nervous system was shown in 41,486 male U.S. physicians who had performed fluoroscopy-guided interventional procedures (FGIP) (exposure assessment: NA) compared to 46,846 male psychiatrists [61]. However, mortality due to degenerative diseases of the nervous system (ICD-9: 331.1–337.9/ICD-10: G31–G32) appeared higher in U.S. radiologists (exposure assessment: NA) compared to the general population (SMR = 1.50; 95% CI: 1.09, 1.90; n_deaths_ = 53) and compared to psychiatrists (RR = 1.39; 95% CI: 0.96, 2.01) even if it was not statistically significant in the latter case [62]. An increased risk of mortality from diseases of the nervous system was found in a cohort of 27,011 medical X-ray workers employed between 1950 and 1980 (average radiation exposure for the workers employed until 1969: 551 mGy; employed between 1970 and 1980: 82 mGy) in China compared to a group of 25,782 non-exposed medical specialists (RR = 2.10; 95% CI: 1.20, 3.10) [64]. On the other hand, a significant decrease in mortality from disease of the nervous system was reported among male diagnostic medical radiation workers (exposure assessment: NA) in South Korea compared with the general population (SMR = 0.54; 95% CI: 0.33, 0.88; n_deaths_ = 16) [60].

#### 3.1.5. Overall Meta-Analyses among Occupational Studies

**Figure 2:**
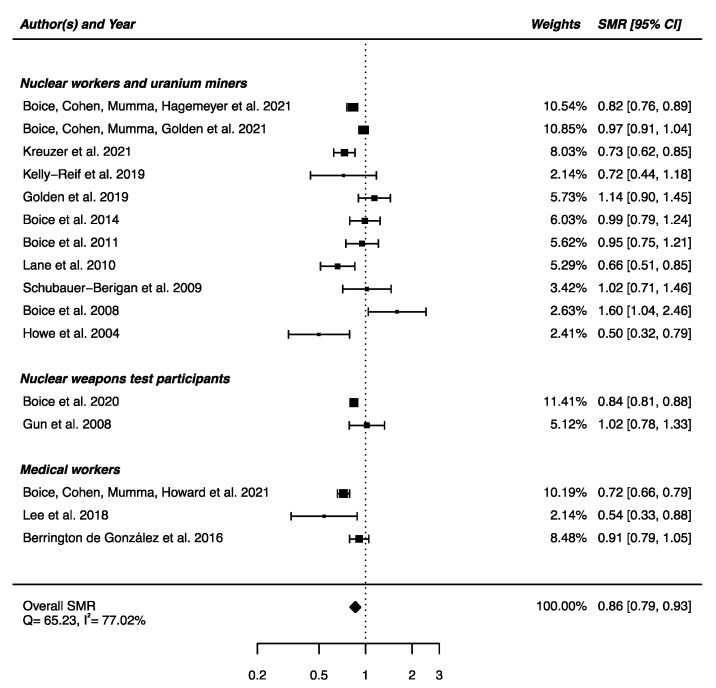
Standardized mortality ratio (SMR) and 95% confidence interval (CI) for mortality from diseases of the nervous system in IR-exposed populations compared with general populations as reference.

An overall SMR was calculated based on the 16 articles presented above. A decrease in mortality due to nervous system diseases was found (SMR_pooled_ = 0.86; 95% CI: 0.79, 0.93), with high heterogeneity between studies (Q = 65.23, *p* < 0.0001) and no publication bias (*p* = 0.91), but with a high percentage of variation across studies that is due to heterogeneity rather than chance (I^2^ = 77.02%) (Figure 2). Sensitivity analyses where studies or different workers groups were excluded one by one were conducted with persistent heterogeneity each time, as well as sensitivity analyses in which studies that did not report ICD codes were removed.

**Figure 3:**
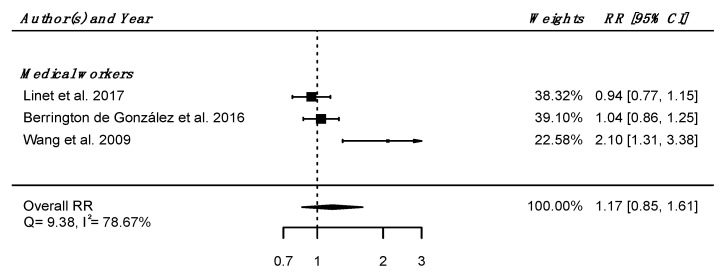
Relative risk (RR) and 95% confidence interval (CI) for mortality from diseases of the nervous system and sense organs in the reviewed studies among IR exposed populations compared with unexposed controls.

Regarding internal comparison, a RR_pooled_ was computed from three studies (all including medical radiation workers [61,62,64]), showing no increased risk of death due to nervous system diseases in IR-exposed people compared to unexposed controls (RR_pooled_ = 1.17; 95% CI: 0.85, 1.61), with high heterogeneity (Q = 9.38, *p* = 0.009), I^2^ = 78.67%, and no suspected publication bias (*p* = 0.21) (Figure 3).

**Figure 4:**
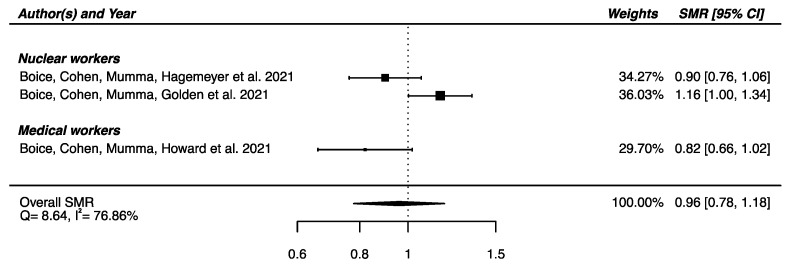
Standardized mortality ratio (SMR) and 95% confidence interval (CI) for mortality from Parkinson’s disease in IR-exposed populations compared with general populations as reference.

Regarding the three studies reporting SMR for Parkinson’s disease [32,33,58] a SMR_pooled_ was computed, showing no significant overall difference in mortality from Parkinson’s disease between the IR-exposed populations presented above and the general populations (SMR_pooled_ = 0.96; 95% CI: 0.78, 1.18), with moderate heterogeneity (Q = 8.64, *p* = 0.013), I^2^ = 78.86% and no publication bias (*p* = 0.34) (Figure 4).

**Figure 5:**
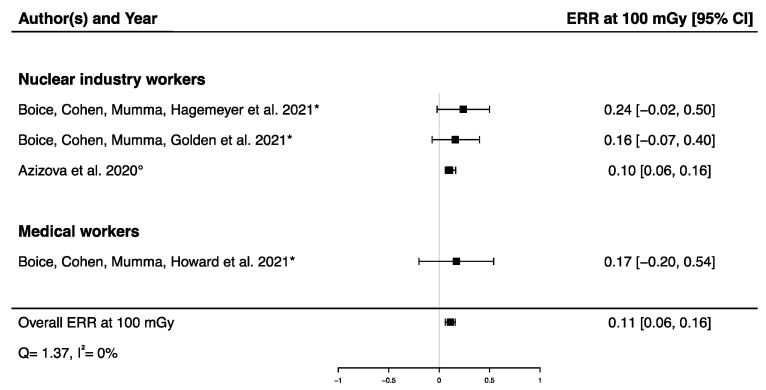
Excess relative risk (ERR) at 100 mGy and 95% confidence interval (CI) for morbidity and mortality from Parkinson’s disease in relation to IR exposure (° incidence; * mortality).

A pooled ERR at 100 mGy from four studies assessing the dose–response relationship between IR exposure and Parkinson’s disease mortality [32,33,58] and incidence [12] was calculated, showing a significant positive ERR (ERR_pooled_ at 100 mGy = 0.11; 95% CI: 0.06, 0.16) with no heterogeneity (Q = 1.37, *p* = 0.71), I^2^ = 0.00%, and no publication bias (*p* = 0.13) (Figure 5). A sensitivity analysis was conducted by performing a meta-analysis containing only mortality data [32,33,58], and the result was consistent with the one presented above (ERR_pooled_ at 100 mGy = 0.19; 95% CI: 0.04, 0.36). It is noted that the ERR in each of the four studies were individually adjusted on sex and age, with a 10-year lag (Table 2).

### 3.2. Cerebrovascular Diseases (ICD-10: I60–I69)

Key findings of the 39 studies focusing on cerebrovascular diseases can be found in (Table 3).

#### 3.2.1. Nuclear Workers and Uranium Miners

Of the 39 studies retrieved that investigated cerebrovascular diseases, 20 focused on nuclear industry workers, of which 5 reported no difference in the mortality of IR-exposed workers compared to the mortality in the reference population [36,37,38,46,47] and 8 reported a decreased mortality compared to general population [32,33,35,40,42,44,45,49], whereas Kreuzer et al. (2021) reported a higher mortality in a cohort of male underground miners compared to the general population (SMR = 1.33; 95% CI: 1.26, 1.41; n_deaths_ = 1335) [34]. When using internal comparison, Gillies et al. (2017) reported a significant positive dose–response relationship for mortality due to cerebrovascular diseases in the international pooled study of radiation workers from the U.K., U.S., and France comprising 308,297 workers (INWORKS cohort) (mean dose: 0.025 Sv) (ERR per Sv = 0.49; 90% CI: 0.11, 0.92; n_deaths_ = 4399) [39]. A significant positive dose–response relationship for mortality due to cerebrovascular diseases was also found in the last update of the U.K. cohort within the INWORKS analyses composed of 166,812 nuclear workers (median dose: 3.1 mSv) (ERR per Sv = 0.57; 95% CI: 0.00, 1.31; n_deaths_ = 3219) [31]. Boice et al. (2021) did not find a statistically significant relationship between cumulative dose and mortality from cerebrovascular diseases among the Los Alamos National Laboratory cohort (ERR at 100 mGy = −0.11; 95% CI: −0.35, 0.12; n_deaths_ = 871) [33]. Azizova et al. (2014) did not report significant associations between external (mean total dose (95% percentile) from external gamma rays: 0.54 Gy (2.21Gy) for men and 0.44 (1.87 Gy) for women) (ERR per Gy = 0.05; 95% CI: −0.03, 0.16; n_deaths_ = 632) nor internal (mean total absorbed alpha-particles dose (95% percentile) to the liver from incorporated plutonium: 0.23 Gy (0.89 Gy) for men and 0.44 Gy (1.25 Gy) for women) (ERR per Gy = 0.17; 95% CI: NA, 0.56; n_deaths_ = 1650) radiation exposure and mortality from cerebrovascular diseases among a cohort of 22,377 Russian Federation Mayak nuclear workers [41]. However, in the last update of the latter cohort, significant positive dose–response relationships were found between external (ERR per Gy = 0.39; 95% CI: 0.31, 0.48) and internal (ERR per Gy = 0.32; 95% CI: 0.16, 0.51) radiation exposure and the incidence of cerebrovascular diseases [30].

In studies on uranium miners, the five retrieved studies yielded dose–response analysis results, and only the French cohort consisting of 5400 workers reported an association between cumulative radon decay products exposure (mean: 35.1 WLM) and cerebrovascular diseases mortality (ERR per 100 WLM = 0.42; 95% CI: 0.04, 1.04; n_deaths_ = 105) [38], whereas the others did not [34,40,45,48].

#### 3.2.2. Nuclear Weapons Test Participants

Regarding nuclear weapons test series, a higher risk of mortality for cerebrovascular diseases was found in 21,357 servicemen and male civilians who participated in the U.K.’s atmospheric nuclear weapon tests and experimental programs compared with 22,312 controls (RR = 1.12; 95% CI: 1.03, 1.21), but mortality in this cohort was not significantly different than in the general population (SMR = 0.91; 95% CI: 0.85, 0.97; n_deaths_ = 816) [52]. Decreased mortality due to cerebrovascular diseases was reported among U.S. military participants compared to the general population (SMR = 0.86; 95% CI: 0.83, 0.89; n_deaths_ = 3161) [53] and among Australian participants to the British nuclear test in Australia compared with the general population (SMR = 0.86; 95% CI: 0.76, 0.98; n_deaths_ = 243) [54].

#### 3.2.3. Chernobyl Cleanup Workers

An increased risk of acute (RR = 1.40; 95% CI: 1.30, 1.50) and chronic (RR = 1.23; 95% CI: 1.00, 1.50) cerebrovascular diseases was shown in 198 Ukrainian Chernobyl liquidators (mean external dose exposure: 456 mSv) compared to 42 controls exposed to <50 mSv [65]. Among 957 evacuees from the 30 km zone of Chernobyl aged 18–60 years at the time of the accident, a significantly increased risk of cerebrovascular diseases was reported in those with a thyroid ^131^I dose of 0.31–0.75 Gy compared to those with a dose below 0.30 Gy (RR = 2.16; 95% CI: 1.45, 3.22) [66]. A statistically significant dose–response relationship was reported between external gamma doses and the incidence of cerebrovascular diseases among 53,772 male Russian Chernobyl emergency workers who arrived in the zone of the Chernobyl accident within the first year after it (26 April 1986–26 April 1987) and who were followed from 1986 to 2012 (mean external whole body dose: 0.161 Gy; max: 1.42 Gy) (ERR per Gy = 0.45; 95% CI: 0.28, 0.62; n_diseases_ = 23,264) [67], whereas the risk of cerebrovascular diseases in an Estonian cohort of Chernobyl cleanup workers was not statistically different from an unexposed comparison cohort of 7631 men (RR = 1.05; 95% CI: 0.91, 1.20) [70].

#### 3.2.4. Flight Attendants

A pooled cohort of 93,771 crew members from 10 countries (exposure assessment: NA) reported a decrease in cerebrovascular diseases mortality among the cockpit crew (SMR = 0.50; 95% CI: 0.41, 0.62; n_deaths_ = 132) and female cabin crew (SMR = 0.47; 95% CI: 0.33, 0.67; n_deaths_ = 45) but not among male cabin crew (SMR = 0.77; 95% CI: 0.53, 1.09; n_deaths_ = 45) compared to that of the general population [55]. Along with the results presented above, Yong et al. showed a decrease in cerebrovascular diseases mortality among 5964 U.S commercial cockpit crew members (pilots and flight engineers) (mean annual cosmic radiation dose: 1.4 mSv (max: 2.8 mSv)) (SMR = 0.61: 95% CI: 0.50, 0.74; n_deaths_ = 108) compared to the general population [56].

#### 3.2.5. Medical Workers

A decrease in mortality due to cerebrovascular diseases was reported in U.S. medical and associated radiation workers (SMR = 0.62; 95% CI: 0.57, 0.68; n_deaths_ = 462) [58], in 80,837 Korean diagnostic medical radiation workers (exposure assessment: NA) (SMR = 0.37; 95% CI: 0.29, 0.49; n_deaths_ = 55; for men only) [60], in U.S. physicians likely to have performed FGIP (exposure assessment: NA) (SMR = 0.42; 95% CI: 0.36, 0.49; n_deaths_ = 173) [61], and among 43,763 U.S. radiologists (exposure assessment: NA) (SMR = 0.52; 95% CI: 0.45, 0.60; n_deaths_ = 242) [62], all compared to general populations. However, when physicians likely to have performed FGIP were compared to psychiatrists, no difference in mortality risk was observed (RR = 0.91; 95% CI: 0.75, 1.09) [61]. Additionally, using an internal comparison, Berrington de González et al. (2016) found a significant decrease in mortality from cerebrovascular diseases in U.S. female radiologists compared to female psychiatrists (RR = 0.28; 95% CI: 0.08, 0.92), but a higher risk of mortality from cerebrovascular diseases among male radiologists compared with male psychiatrists in the category of graduates before 1940 (RR = 1.49; 95% CI: 1.11, 2.01) [62]. An increased mortality risk due to cerebrovascular diseases was also observed among X-ray workers in China compared to non-exposed medical specialists (RR = 1.40; 95% CI: 1.20, 1.50) [64]. Furthermore, Rajaraman et al. (2016) observed a 34% increase in stroke incidence in technologists who performed FGIP procedures (exposure assessment: NA) compared to those who never performed these procedures (HR = 1.34; 95% CI: 1.10, 1.64), but no impact on stroke mortality was reported (HR = 1.22; 95% CI: 0.85, 1.73) [63]. However, no significant dose–response relationships between occupational IR exposure and cerebrovascular diseases mortality among U.S. medical radiation (ERR at 100 mGy = 0.04; 95% CI: −0.16, 0.23; n_deaths_ = 462) [58], nor between occupational IR exposure and cerebrovascular disease incidence among 11,500 Korean diagnostic medical radiation workers (mean cumulative heart dose: 10.2 mGy) (ERR at 100 mGy = 3.10; 95% CI: −0.75, 11.59; n_diseases_ = 109), were reported [59].

#### 3.2.6. Medical Patients

In the only available study on patients, there was no significantly increased risk of cerebrovascular diseases mortality among a cohort of 77,275 tuberculosis patients in Canada and Massachusetts according to X-ray fluoroscopic diagnostic repeated exposures (ERR per Gy = 0.441; 95% CI: −0.119, 1.090; n_deaths_ = 1561 for cumulative doses restricted to 0–0.5 Gy) [73].

#### 3.2.7. Atomic Bomb Survivors

A statistically significant positive dose–response relationship was found between weighted colon doses (radiation doses from 0 to >3 Gy, 86% received <0.2 Gy) and death from stroke in the 86,611 Life Span Study cohort members (ERR per Gy = 0.09; 95% CI: 0.01, 0.17; n_deaths_ = 9622) [51].

#### 3.2.8. Environmental Radiation

Only one study among those included in this review focused on indoor radon exposure, and it found a slight but significant association with indoor radon level towards an increased incidence of stroke among a South Korean cohort of 28,557 selected inhabitants based on demographic criteria and aged over 40 years (OR = 1.004; 95% CI: 1.001, 1.007) [57].

#### 3.2.9. Overall Meta-Analyses

**Figure 6:**
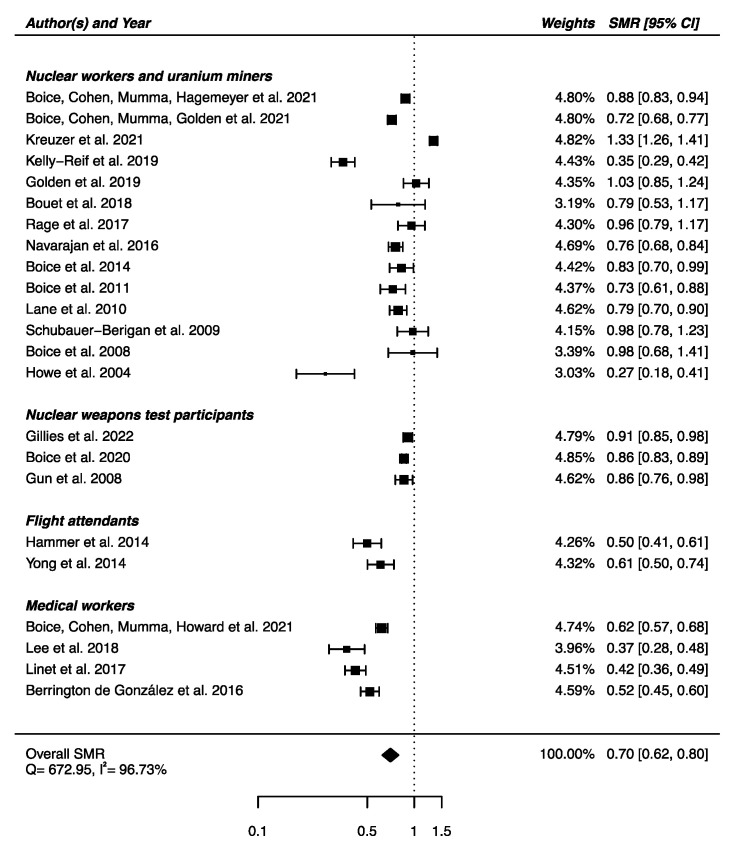
Standardized mortality ratio (SMR) and 95% confidence interval (CI) for mortality from cerebrovascular diseases in IR exposed populations compared with general populations as reference.

From the 23 studies reporting SMRs, an SMR_pooled_ was computed showing a statistically significant lower mortality from cerebrovascular diseases in IR-exposed populations compared with general populations (SMR_pooled_ = 0.70; 95% CI: 0.62, 0.80), with high heterogeneity (Q = 672.95, *p* < 0.0001), I^2^ = 96.73%, and a publication bias (*p* = 0.03), suggesting that small studies with negative results were published less often (Figure 6). Sensitivity analyses where studies or different worker groups were excluded one by one resulted in no change in heterogeneity, as well as sensitivity analyses in which studies that did not report ICD codes were removed.

**Figure 7:**
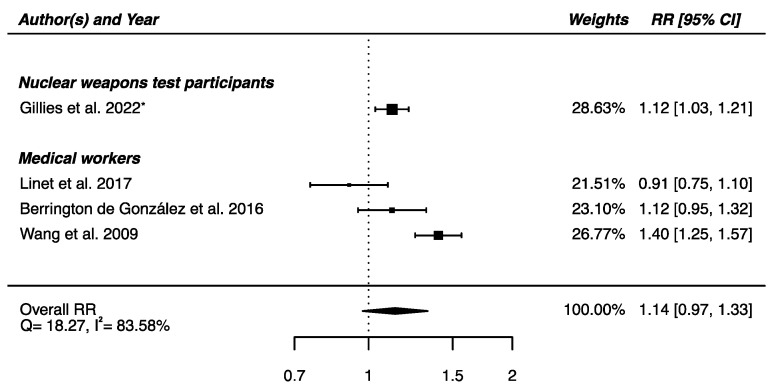
Relative risk (RR) and 95% confidence interval (CI) for mortality from cerebrovascular diseases in the reviewed studies among IR exposed populations compared with unexposed controls. * 90% CI.

The meta-analysis performed on the four studies with available RRs (one on nuclear weapons test participants [52] and the others on medical workers [61,62,64]) yielded a non-significant higher risk of death from cerebrovascular diseases in IR-exposed populations compared with that of unexposed controls (RR_pooled_ = 1.14; 95% CI: 0.97, 1.33), showing heterogeneity (Q = 18.27, *p* = 0.0004), I^2^ = 83.58%, and no suspected publication bias (*p* = 0.69) (Figure 7).

**Figure 8:**
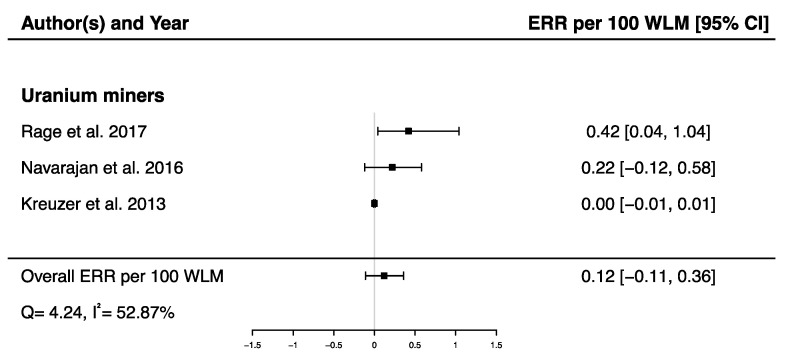
Excess relative risk (ERR) per 100 WLM and 95% confidence interval (CI) for mortality from cerebrovascular diseases in relation to IR exposure.

A pooled ERR per 100 WLM from three studies assessing the dose–response relationship between radon exposure and death from cerebrovascular diseases [38,40,43] was calculated showing a non-significant ERR (ERR_pooled_ per 100 WLM = 0.12; 95% CI: −0.11, 0.36), with moderate heterogeneity (Q = 4.24, *p* = 0.12), I^2^ = 52.87%, and no publication bias (*p* = 0.09) (Figure 8).

**Figure 9:**
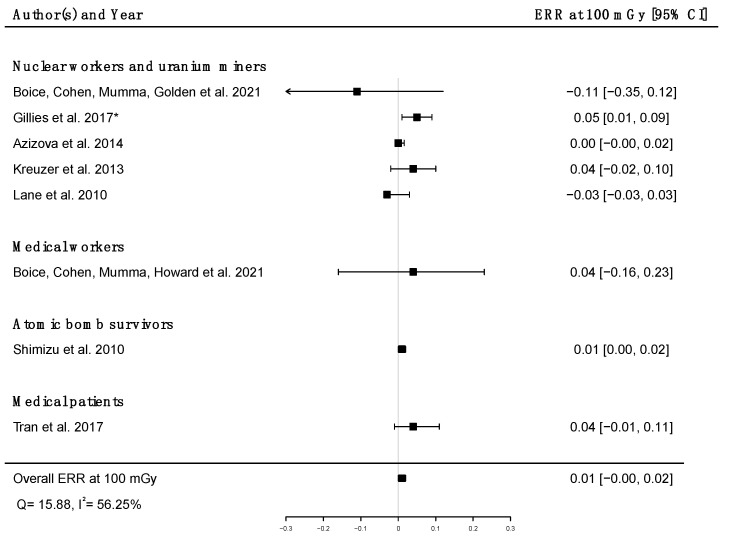
Excess relative risk (ERR) at 100 mGy and 95% confidence interval (CI) for mortality from cerebrovascular diseases in relation to IR exposure; * 90%CI.

**Figure 10:**
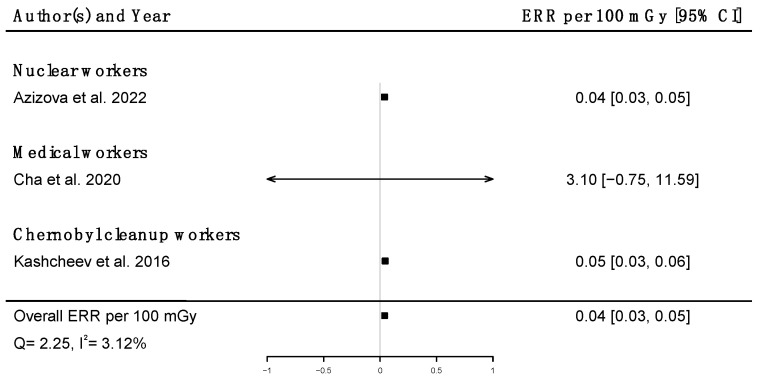
Excess relative risk (ERR) per 100 mGy and 95% confidence interval (CI) for morbidity from cerebrovascular diseases in relation to IR exposure.

Pooled dose–response relationships between IR and mortality [33,39,41,43,45,51,58,73] or incidence [30,59,67] from cerebrovascular diseases were computed. A positive but non-significant ERR was found for mortality (ERR_pooled_ at Gy = 0.01; 95% CI: −0.00, 0.02; Q = 16.00, (*p* = 0.03), I^2^ = 56.23%, no publication bias (*p* = 0.84) (Figure 9), whereas a statistically significant ERR was obtained for morbidity (ERR_pooled_ per 100 mGy = 0.04; 95% CI: 0.03, 0.05; Q = 2.06 (*p* = 0.36), I^2^ = 3.12%, publication bias (*p* = 0.02)) (Figure 10). However, the estimated ERR at 100 mGy for mortality from cerebrovascular diseases became significant in sensitivity analyses where studies that did not report ICD codes were removed (i.e., the Eldorado uranium workers cohort study [45]) (ERR_pooled_ at 100 mGy = 0.13; 95% CI: 0.03, 0.22).

### 3.3. Mental and Behavioral Disorders (ICD-10: F00–F99)

Key findings of the 22 studies focusing on mental and behavioral disorders can be found in Table 4.

#### 3.3.1. Nuclear Workers and Uranium Miners

Nuclear industry workers were the subject of 12 studies out of the 22 dealing with mental and behavioral disorders. Six of them reported no statistical difference in mortality [33,36,42,44,46,47] compared with general populations. Decreases in deaths due to mental and behavioral disorders were reported among U.S. nuclear power plant workers (SMR = 0.77; 95% CI: 0.70, 0.85; n_deaths_ = 425) [32], among underground miners (SMR = 0.81; 95% CI: 0.70, 0.94; n_deaths_ = 191) [34], and among Eldorado uranium workers (SMR = 0.44; 95% 0.29, 0.63; n_deaths_ = 29) [45] compared with general populations, whereas an increase was reported among 16,434 male uranium miners in the Czech Republic (exposure assessment: NA) (SMR = 1.88; 95% CI: 1.05, 2.71; n_deaths_ = 20) compared to the general population [35]. On the other hand, a statistically significant positive dose–response relationship was found between brain dose and death from mental and behavioral disorders in the INWORKS cohort (ERR per Sv = 1.30; 90% CI: 0.23, 2.72; n_deaths_ = 705) [39]. Additionally, an increased risk of death from dementia was found in a nested case–control study within a pooled cohort of 67,976 female nuclear workers occupationally exposed to IR, including 91 cases and 910 controls of which 14 cases and 154 controls were monitored for radiation (max annual radiation dose: 49.9 mSv) (OR = 2.09; 95% CI: 1.02, 4.29) [50].

#### 3.3.2. Nuclear Weapons Test Participants

Among U.S. military participants in nuclear weapons test series, a significant decrease in mortality due to mental and behavioral disorders was reported as compared with the general population (SMR = 0.92; 95% CI: 0.87, 0.98; n_deaths_ = 1021) [53].

#### 3.3.3. Chernobyl Cleanup Workers

A statistically significant dose-dependent increase in the level of mental disorders (as assessed by the Brief Psychiatric Rating Scale (BPRS) [74]) was found among 326 Ukrainian cleanup workers exposed to dose under 500 mSv [69]. Higher risks of organic psychoses and non-psychotic organic brain damages were found among 198 Ukrainian Chernobyl liquidators who intervened in 1986–1987, relative to 42 internal controls exposed to doses < 50 mSv (RR = 3.15; 95% CI: 2.60, 3.70 and RR = 1.99; 95% CI: 1.60, 2.50, respectively) [65]. Regarding schizophrenia spectrum disorders, the incidence increased dramatically among 100 Chernobyl exclusion zone personnel with acute radiation sickness as compared to the general Ukrainian population in 1990, just after the disaster (5.4/10,000 vs. 1.1/10,000, respectively) [72].

Furthermore, a statistically significant increased frequency of mild cognitive disorders was observed among 196 men workers involved in the Chernobyl “Shelter Object” (total irradiation mean: 19.9 mSv; max: 56.7 mSv) between baseline (T0) and after completion of their period of work on-site (T1) (3.6% vs. 11.2% (*p* < 0.01), respectively). Nevertheless, this increase was not found in workers who had already been exposed to IR before this task [68]. Loganovsky et al. (2013) found a higher level of depression assessed by the self-rating depression scale [75] in 219 people with post-traumatic stress disorder (PTSD) affected by the Chernobyl disaster, whether they were diagnosed with acute radiation sickness (mean and standard deviations: 52.3 ± 12.9) or not (58.6 ± 12.6), compared with 28 war veterans (47.8 ± 12.6) and a group of 22 healthy unexposed people (39.6 ± 7.3) [71]. On the other hand, no increase in the incidence of mental and behavioral disorders was found in Estonian Chernobyl clean-up workers compared to a cohort of unexposed men (RR = 1.00; 95% CI: 0.94, 1.07) but was found for mental disorders due to alcohol (RR = 1.21; 95% CI: 1.06, 1.39) [70].

#### 3.3.4. Medical Workers

Among medical workers, a lower risk of mortality due to mental and behavioral disorders was reported among U.S. medical radiation workers (SMR = 0.58; 95% CI: 0.51, 0.66; n_deaths_ = 246) compared with the general population [58]. Furthermore, a lower risk of mortality was reported among U.S. physicians likely to have performed FGIP compared with psychiatrists (RR = 0.55; 95% CI: 0.35, 0.84) [61], whereas a non-significant higher risk was shown among male radiologists compared to psychiatrists (RR = 1.30; 95% CI: 0.60, 2.80) [62]. In this latter study, a decreased mortality was shown among male radiologists compared with national rates (SMR = 0.51; 95% CI: 0.38, 0.64, n_deaths_ = 60) [62].

#### 3.3.5. Overall Meta-Analysis

**Figure 11:**
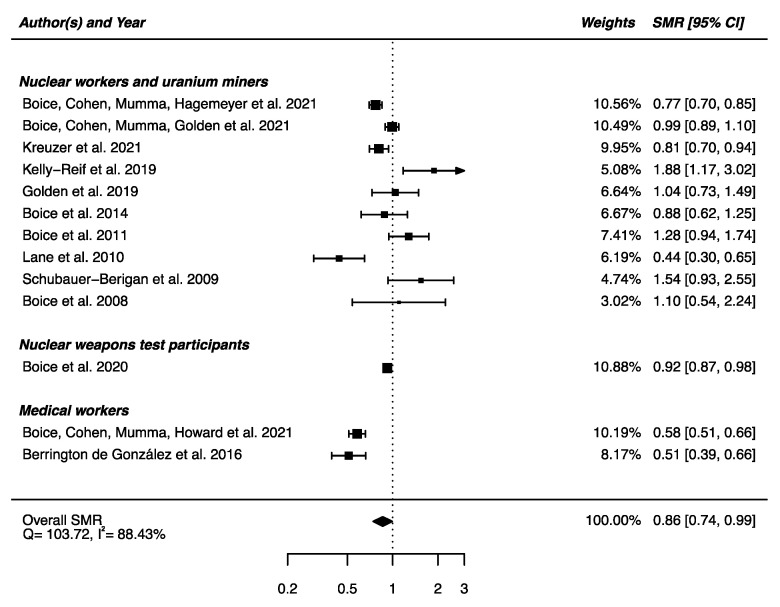
Standardized mortality ratio (SMR) and 95% confidence interval (CI) for mortality from mental and behavioral disorders in IR exposed populations compared with general populations as reference.

Out of the 22 studies included in this section, 13 had available SMRs to be integrated in a meta-analysis, showing a statistically significant lower mortality from mental and behavioral disorders between the IR-exposed populations presented above and the general populations (SMR_pooled_ = 0.86; 95% CI: 0.74, 0.99, Q = 103.72 (*p* < 0.0001), I^2^ = 88.43%, no publication bias (*p* = 0.97) (Figure 11). Sensitivity analyses where studies or different workers groups were excluded one by one resulted in no change in heterogeneity, as well as sensitivity analyses in which studies that did not report ICD codes were removed.

## 4. Discussion

The risk of non-cancerous CNS diseases after exposure to low-to-moderate doses of IR in adulthood was analyzed based on 45 studies. Meta-analyses show reduced mortality due to nervous system diseases, cerebrovascular diseases, and mental and behavioral disorders in radiation workers compared to general populations and suggest a higher risk of cerebrovascular diseases and Parkinson’s disease that may be dose-dependent in people exposed during adulthood (radiation workers, A-bomb survivors). For cerebrovascular diseases, a significant dose-risk relationship is reported for incidence, but it was non-significant for mortality. These findings are consistent with the previous meta-analysis by Little et al. (2012), who found a significant positive relationship between cumulative IR dose and cerebrovascular diseases, studying mortality and morbidity outcomes together [13]. We conducted separate analyses for mortality and incidence outcomes because mortality analyses are often based on the underlying cause-of-death, and it has been shown that cerebrovascular diseases could be poorly captured when using only underlying causes of death [76].

In the present study, the decreases in mortality found in radiation workers when compared to general population rates for nervous system diseases, cerebrovascular diseases, and mental and behavioral diseases can be explained by the healthy-worker effect [77], meaning that worker populations usually present a better health condition than the general population. The calculation of relative risks using a control occupational group that is supposed to be more comparable to the exposed group (except for exposure) allows the healthy-worker effect to be avoided. This can be observed with the pooled analysis of the results of one study on nuclear weapons test participants [52] and three studies of medical radiation workers compared to groups of unexposed populations [61,62,64] that yields nonsignificantly increased relative risks of mortality from nervous or cerebrovascular diseases. Internal dose–risk analyses are even more informative for investigating the impact of IR exposure on the risk of non-cancerous CNS diseases.

Most of the studies included in this review dealt with occupational exposure, mainly among nuclear workers and uranium miners, who are subject to different types of exposure depending on their activities. For example, uranium miners are repeatedly exposed to a mix of radon gas and its progenies, external gamma rays, and uranium dusts [78], and cycle nuclear workers are exposed to external gamma rays, possibly combined with tritium, uranium, or plutonium, depending on their activity [79,80]. However, external exposures are more commonly reported in studies, although internal contamination was often mentioned among nuclear workers. Due to the low rate of workers monitored for internal exposure, or the fact that only the status “exposed to internal contamination” was known [39], few studies performed separate exposure-based, adjusted, or sensitivity analyses to disentangle the share of risk attributable to each type of exposure [30,36,41,42,44], which did not allow specific meta-analyses for internal exposures in this work. Nevertheless, some studies have addressed the issue of co-exposure by treating it as a confounding factor [41], and it did not significantly change the result (ERR per Gy = 0.05; 95% CI: −0.03, 0.16 vs. ERR per Gy = > 0; 95% CI: −0.10, 0.16). In addition, differences in results between studies may be related to the characteristics of the exposure such as brief or prolonged; the type of radiation field (e.g., external low-LET photons vs. high-LET alpha particles); or possible biases in dosimetry [32].

Furthermore, exposure to IR in the occupational setting is often accompanied by co-exposure to other health risk factors (e.g., chemical substances, pesticides, heavy metals, nitro compounds, non-ionizing radiations, air pollution, tobacco use, etc.) that may confound and/or modify the relationship between IR exposure and a health outcome. For example, medical radiation workers are predominantly exposed to X-rays, but can possibly be exposed to chemicals or drugs (such as hydroquinone, aldehydes, acetic acid, ammonia, etc.) [61,81]. Nevertheless, research on the health effects of co-exposures to two or more risk factors (exposome) is a very dynamic area of research, and synergies or antagonisms following co-exposure to different environmental agents have been shown [82,83]. However, the interaction of various factors and associated health outcomes are poorly characterized to date. In the present work, few studies have considered co-exposures, with limited evidence on their impact on the dose–response relationship.

A high number of risk factors for non-cancerous CNS diseases have been identified in the scientific literature, with varying degrees of evidence depending on the outcome [84,85]. Several risk factors have been considered in the dose–response analyses by each study separately. However, socio-economic factors (often used in occupational studies to control for possible confounding factors that are not available on an individual basis and may influence mortality and disease occurrence) were included in only half of the studies that report dose–response analyses in the present work (Table 2, Table 3 and Table 4). In order to take risk factors into account as much as possible, we performed our meta-analysis on adjusted estimates even if the estimates were not systematically adjusted on the same risk factors, as is usually recommended in meta-analysis methodology.

Finally, mental and behavioral disorders are known to be influenced by individual characteristics, but also by the socio-economic and environmental circumstances in which people live [86]. Indeed, specific disorders such as Alzheimer’s disease and dementia spectrum disorders are known to be influenced by environmental and/or genetic factors [87,88]. It is therefore important to highlight that disasters (natural and human-made) can inflict psychological damage on the affected populations. It has been reported that a major health impact of the Chernobyl nuclear power plant accident was the fear about potential upcoming health problems [89]. Furthermore, the Hiroshima and Nagasaki bombings have had long-lasting effects on mental health, such as post-traumatic stress, depression, anxiety, and somatization. However, there were few well-designed studies (i.e., evaluation of exposure, confounding factors) on mental health following the Chernobyl catastrophe. It appears very difficult to determine which part of mental health disorders is due to radiation directly, and which is due to the psychological consequence of having experienced such a disaster [90]. In a broader sense, when studying non-cancerous CNS diseases, such as cognitive disorders, many factors such as exposure time vulnerability, mechanism, and susceptibility factors are important to consider [11].

One major limitation of this systematic review was related to the definition of outcomes. Although most of the studies are based on the ICD coding, whether for causes of death or diagnostics (which still ensures a certain homogeneity and reliability in our analyses), the fact remains that classification errors may have been made during coding. For example, the rules of the ICD lead to the selection of suicide as the initial cause of death, even if the physician has indicated another sequence (e.g., depression leading to suicide) [91]. Thus, the number of deaths due to depression may have been underestimated in these studies. In Russia, a high degree of inconsistency across the region was found for mental and behavioral disorders, diseases of the nervous system, and certain cardiovascular diseases, suggesting differences in coding practices [92]. Then, analyses could be performed on broad categories, as the level of consistency improves when causes of death are grouped into broader diagnostic categories, but could not be performed for subcategories, when the classification bias might be higher. Finally, 10 studies did not even mention ICD coding, which has occasionally made it difficult to classify the outcomes of these studies within the causes of disease/death used in this work, possibly leading to a classification bias. Sensitivity analyses performed by removing the studies without ICD codes showed no change in the results, except the positive dose–response relationship for cerebrovascular diseases, which became significant.

Heterogeneity seems unavoidable because of different populations, various types of radiation exposure, and chronical or acute exposure. Sensitivity analyses in which the pooled SMR was calculated excluding each study one at a time and each group (e.g., aircrews, nuclear workers, medical workers, etc.) revealed no substantial alteration of the aggregate SMR for the three studied outcomes. Nevertheless, we used random effects models to calculate our estimates (SMR, RR, and ERR), which account for potential heterogeneity between and within studies.

The consideration of bioindicators and biomarkers in epidemiological studies could be very informative in improving the accuracy of the outcomes and the reconstruction of actual IR exposure of participants. In the long run, this could also help to better understand the mechanisms of these neurodegenerative disorders. For example, Borghini et al. (2017) have shown that the expression profiles of circulating brain miR-134 (a brain-specific miRNA that has been shown to be dysregulated in pathologies such as Alzheimer disease, bipolar disorder, and glioblastomas [93]) and miR-2392 were significantly downregulated in interventional cardiologists compared with controls [94]. Complementary studies are needed to confirm these findings and to further explore the potential of circulating miRNAs to be used clinically as novel biomarkers to identify early, disease-related perturbations caused by long-term radiation exposure.

According to this work, the effects of low doses of IR on non-cancerous CNS diseases cannot be excluded. Compared to the 2.7 billion people who had neurological disorders in 2019 [9], the estimated increased risk of 17% would result in a significant public health impact. In addition, due to the fact that human populations are increasingly exposed to IR from various sources (e.g., cosmic rays, environmental radionuclides), along with the continued growth and evolution of IR imaging technologies, the resulting dose in the general population is increasing. Moreover, in a context where exposure to IR is steadily increasing in some groups of workers (e.g., medical radiation workers), new studies avoiding the biases mentioned in this work are justified: the use of precise dosimetry, an indisputable definition of the outcomes, and adjusted dose–response calculations are encouraged. To our knowledge, this work is the first systematic review and meta-analysis of the literature assessing the risk of non-cancerous CNS diseases and mortality in populations exposed to IR during adulthood only. We included a broad range of endpoints, resulting in a large number of studies covered. All included studies met the previously defined criteria according to the PRISMA recommendations, allowing robust and exhaustive analysis while maintaining a focus on the main research question. The quality score between 4 and 9 on the Newcastle Ottawa scale for all studies included in this review provides a good quality rating for this work.

## 5. Conclusions

The present review and meta-analyses did not suggest higher risk of mortality due to non-cancerous CNS diseases after adult IR-exposure compared with unexposed controls. However, some of the studies reviewed had low statistical power to detect an effect and inadequate dosimetry, if any. Furthermore, a significant positive excess relative risk was found for cerebrovascular disease morbidity and for Parkinson’s disease. Nevertheless, we caution against drawing firm conclusions due to methodological issues, including uncertainties related to the classification of the diseases, dosimetry assessment, and potential confusion bias. Further studies, ideally large-scale studies with individual dose reconstruction and collection of information on potential confounding factors, will be essential to expand our knowledge of the risk of non-cancerous CNS diseases following exposure to low-dose IR.

## Figures and Tables

**Table 1:** Characteristics of the included studies.

First Author, Year	Country	Design	Population	Exposure Assessment	NOS Scores
***Nuclear workers and uranium miners***					
**Azizova et al., 2022** [30]	Russia	Cohort	22,377 Russia Mayak nuclear workers (M/F)	Mean cumulative liver absorbed dose of gamma rays from external exposure: 0.45 (SD: 0.65) Gy (M), 0.37 (SD: 0.56) Gy (F) Mean alpha activity: 1.20 (SD: 4.42) kBq (M), 1.83 (SD: 10.03) kBq (F) Mean cumulative liver-absorbed doses of alpha particles from internal exposure: 0.18 (SD: 0.65) Gy (M), 0.40 (SD: 1.92) Gy (F)	8
**Hinksman et al., 2022** [31]	U.K.	Cohort	150,390 (M), 16,422 (F) radiation workers	Median dose (IQR): 3.1 (0.3, 16.0) mSv	8
**Boice, Cohen, Mumma, Hagemeyer et al., 2021** [32]	USA	Cohort	130,773 (M), 4420 (F) nuclear power plant workers	Mean dose to the brain: 33.2 mGy (max: 0.83 Gy)	8
**Boice, Cohen, Mumma, Golden et al., 2021** [33]	USA	Cohort	19,808 (M), 6520 (F) workers at the Los Alamos National Laboratory	Brain radiation absorbed dose, combining external and internal sources for Pu: mean: 11.6 mGy; median: 0.76 mGy; max: 760 mGy	8
**Kreuzer et al., 2021** [34]	Germany	Cohort	35,204 (M) underground miners	Mean cumulative exposure to radon: 364 WLM and silica dust: 7.6 mg/m^3^ -years	8
**Azizova et al., 2020** [12]	Russia	Cohort	16,688 (M), 5689 (F) Mayak workers	Mean cumulative dose from external gamma-rays absorbed in the brain: 0.46 ± 0.67 (95th percentile: 1.55 Gy, min-max: 0.00–8.01 Gy) (M), 0.36 ± 0.56 Gy (95th percentile: 1.55, min-max: 0.00–6.14 Gy) (F)	9
**Kelly-Reif et al., 2019** [35]	Czech Republic	Cohort	16,434 (M) underground uranium miners	NA	7
**Golden et al., 2019** [36]	USA	Cohort	2514 (M) Mallinckrodt uranium processing workers	Mean brain dose from all sources of external and internal radiation combined: 37.2 mGy (max: 750 mGy)	8
**Bouet et al., 2018** [37]	France	Cohort	1180 (M), 111 (F) uranium millers	NA	7
**Rage et al., 2017** [38]	France	Cohort	5400 (M) uranium miners	Cumulative exposure (WLM), mean (se): 35.1 (69.9), median (min-max): 10.8 (0.002–960.1)	7
**Gillies et al., 2017** [39]	France, U.K. and USA	Joint analysis cohorts	308,297 (M/F) nuclear workers	Average cumulative dose: 25.2 mSv, collective dose: 7771.5 Sv, median dose: 3.4 mSv, 90th percentile dose: 64.5 mSv, max dose: 1932 mSv	8
**Navaranjan et al., 2016** [40]	Canada	Cohort	28,546 (M), 413 (F) uranium miners	Mean cumulative exposure: 21.0 WLM, range: 0.0–875.1 WLM (M) and 0.2 WLM, range: 0.0–16.3 WLM (F)	8
**Azizova et al., 2014** [41]	Russia	Cohort	16,688 (M), 5689 (F) Mayak workers	Mean (±SD) total dose from external gamma rays: 0.54 ± 0.76 Gy (95% percentile 2.21 Gy) (M) and 0.44 ± 0.65 Gy (95% percentile 1.87 Gy) (F). Mean plutonium body burden: 1.32 ± 4.87 kBq (95% percentile 4.71 kBq) (M) and 2.21 ± 13.24 kBq (95% percentile 4.56 kBq) (F). Mean total absorbed alpha-particles dose to the liver from incorporated plutonium: 0.23 ± 0.77 Gy (95% percentile 0.89 Gy) (M) and 0.44 ± 2.11 Gy (95% percentile 1.25 Gy) (F)	7
**Boice et al., 2014** [42]	USA	Cohort	4004 (M), 973 (F) mound workers	Mean dose from external radiation: 26.1 mSv (max: 939.1 mSv). Mean lung dose from internal exposure: 100.1 mSv (max: 17.5 Sv). Mean liver dose from external and internal radiation: 34.6 mSv (max: 2.3 Sv)	7
**Kreuzer et al., 2013** [43]	Germany	Cohort	58,982 (M) uranium miners (WISMUT cohort)	Mean cumulative gamma dose: 47 mSv (max: 909 mSv) Mean cumulative exposure to radon progeny or long-lived radionuclides: 280 WLM (max = 3224) and 4.1 kBqh/m^3^ (max = 132), respectively	8
**Boice et al., 2011** [44]	USA	Cohort	5335 (M), 466 (F) radiation workers at Rocketdyne	Mean dose from external radiation: 13.5 mSv (max: 1 Sv) and the mean lung dose from external and internal radiation combined: 19.0 mSv (max: 3.6 Sv)	7
**Lane et al., 2010** [45]	USA	Cohort	16,236 (M) and 1424 (F) uranium workers (Eldorado cohort)	Mean radon decay products exposure (SD): 100.2 WLM (254.4 WLM) (M), 4.6 WLM (10.1 WLM) (F); mean gamma-ray dose (SD): 52.2 mSv (152.4 mSv) (M), 34.4 mSv (77.4 mSv) (F)	8
**Schubauer-Berigan et al., 2009** [46]	USA	Cohort	3358 white uranium miners (M) and 779 uranium miners of another race (M) (Colorado Plateau Cohort)	Cumulative radon exposure: from uranium mining: mean (SD): 806 WLM (1130 WLM); median (interdecile interval): 426 WLM (44.1–2070 WLM) for whites and mean (SD): 742 WLM (840 WLM); median (interdecile interval): 392 WLM (43.7–2010 WLM) for American Indians and including hard rock mines: mean (SD): 824 WLM (1140 WLM); median (interdecile interval): 439 WLM (55.0–2080 WLM) for whites and mean (SD): 742 WLM (840 WLM); median (interdecile interval): 392 WLM (43.7–2030 WLM) for American Indians	8
**Boice et al., 2008** [47]	USA	Cohort	2500 (M), 245 (F) uranium millers and miners	NA	7
**Villeneuve et al., 2007** [48]	Germany	Cohort	2070 (M) miners (Newfoundland fluorspar cohort)	Mean annual radon exposure among the underground miners: 43.6 WLM/year	8
**Howe et al., 2004** [49]	USA	Cohort	47,311 (M), 6387 (F) nuclear power industry workers	Mean cumulative dose: 28.5 mSv (M), 4.6 mSv (F) and 25.7 mSv for the all cohort	7
**Sibley et al., 2003** [50]	USA	Case-control	91 cases and 910 controls (F) nuclear weapons workers	14 cases and 154 controls monitored for radiation. Max annual radiation dose: 49.9 mSv	6
***Atomic bomb survivors***					
**Shimizu et al., 2010** [51]	Japan	Cohort	35,687 (M), 50,924 (F) Hiroshima and Nagasaki atomic bomb survivors (Life Span Study cohort)	Individual estimate radiation doses ranged from 0 to >3 Gy (86% of the cohort members received <0.2 Gy)	8
***Nuclear weapons test participants***					
**Gillies et al., 2022** [52]	U.K.	Cohort	21,357 servicemen and male civilians from the U.K. who participated in the U.K.’s atmospheric nuclear weapon tests and experimental programs and a group of 22,312 controls	8% of the total participant cohort had non-zero recorded radiation doses and the mean dose from gamma radiation amongst these men was 9.9 mSv	7
**Boice et al., 2020** [53]	USA	Cohort	114,270 (M) military participants at eight aboveground nuclear weapons test series	Mean NuTRIS film badge gamma radiation dose: 6 mSv (max: 908 mSv)	8
**Gun et al., 2008** [54]	Australia	Cohort	10,983 (M) participants in the British nuclear tests in Australia	Total (collective) dose was approximately 31 Sv	8
***Flight attendants***					
**Hammer et al., 2014** [55]	10 Countries	Joint analysis of cohorts	12,288 (M), 44,667 (F) cabin crew and 36,816 (M) cockpit crew	NA	7
**Yong et al., 2014** [56]	U.S.A.	Cohort	5958 (M), 6 (F) commercial airline cockpit crew	Mean annual cosmic radiation dose: 1.4 mSv (median: 1.4 mSv, range: 0.0042–2.8 mSv)	8
***Environmental exposure***					
**Kim et al., 2020** [57]	South Korea	Cross-sectional study	12,154 (M), 16,403 (F) study participants	Radon exposure: 103.1 ± 22.0 Bq/m^3^	8
***Medical workers***					
**Boice, Cohen, Mumma, Howard et al., 2021** [58]	USA	Cohort	55,218 (M), 53,801 (F) medical and associated radiation workers	Mean (max) cumulative absorbed dose to the brain: 18.9 mGy (1.08 Gy)	8
**Cha et al., 2020** [59]	South Korea	Cohort	7827 (M), 3673 (F) medical radiation workers	Mean cumulative badge dose: 7.20 mSv. Mean cumulative heart dose: 6.2 mGy (range: 0.002–72.9 mGy)	8
**Lee et al., 2018** [60]	South Korea	Cohort	80,837 (M/F) diagnostic medical radiation workers	NA	7
**Linet et al., 2017** [61]	USA	Cohort	Physicians likely to perform fluoroscopy guided interventional procedures (*n* = 41,486 (M), *n* = 4148 (F). Psychiatrists (*n* = 46,846 (M), 17,555 (F))	NA	7
**Berrington de González et al., 2016** [62]	USA	Cohort	Radiologists (*n* = 34, 912 (M), *n* = 8851 (F)). Psychiatrists (*n* = 47,497 (M), *n* = 17,493 (F))	NA	7
**Rajaraman et al., 2016** [63]	USA	Cohort	65,131 (M), 19,835 (F) radiologic technologists (U.S. Radiologic Technologists cohort)	NA	8
**Wang et al., 2009** [64]	China	Cohort	21,586 (M), 5443 (F) medical diagnostic X-ray workers compared to 17,694 (M), 8088 (F) other medical specialists	Average radiation exposure for the workers employed until 1969: 551 mGy; employed between 1970 and 1980: 82 mGy	7
***Chernobyl cleanup workers***					
**Loganovsky et al., 2020** [65]	Ukraine	Cohort and cross-sectional study	198 Chernobyl clean-up workers compared to 110 men. Internal comparison group: 42 clean-up workers irradiated at dose 0.6–50.0 mSv	Average dose of the external exposure of the examined clean-up workers: 456.0 mSv (SD: 760.0 mSv), range: 0.6–5900.0 mSv	6
**Buzunov et al., 2018** [66]	Ukraine	Cohort	18,133 (M), 24,849 (F) evacuees from the Chernobyl zone	Personnel data on radiation dose are available for: 957 people. Dose intervals: 0–0.3 Gy, 0.31–0.75 Gy, 0.76–2.0 Gy, above 2 Gy	6
**Kashcheev et al., 2016** [67]	Russia	Cohort	53,772 (M) recovery operation workers of the Chernobyl accident	Mean external whole body dose: 0.161 Gy (max: 1.42 Gy)	7
**Loganovsky et al., 2016** [68]	Ukraine	Cohort	196 men examined before (t0) and after (t1) working on the Shelter Object (SO)	In the observational period, the SO staff were exposed to external irradiation at the dose range of 0–54.3 mSv (mean ± SD: 19.5 ± 12.8 mSv), to internal irradiation at the dose range of 0–2.4 mSv (mean ± SD: 0.4 ± 0.5 mSv), and to total irradiation at the dose range of 0–56.7 mSv (mean ± SD: 19.9 ± 13.0 mSv)	5
**Bazyka et al., 2015** [69]	Ukraine	Cohort	326 Chernobyl cleanup workers, 290 of which had doses under 500 mSv. Internal control group: 44 other workers exposed to doses less than 20 mSv.	Radiation dose values ranged from 0.8 mSv to 2800 mSv (274.0 ± 418.9 mSv) (mean ± SD)	6
**Rahu et al., 2014** [70]	Estonia	Cohort	3680 exposed cleanup workers from Estonia compared to an unexposed cohort of 7631 men	Mean and median radiation doses: 9.9 and 8.9 cGy respectively (range: 0.0–54.5 cGy)	7
**Loganovsky et al., 2013** [71]	Ukraine	Cross-sectional study	241 people, 219 of whom have been diagnosed with PTSD: 115 cleanup workers of the Chernobyl accident (34 with ARS), 76 evacuees from the Chernobyl exclusion zone. Comparison group: 28 veterans of the war of Afghanistan. Control group: 22 healthy unexposed individuals.	Cleanup workers without ARS: dose range of 3.1–856.0 mSv (mean ± SD: 247.2 ± 224.1 mSv) Cleanup workers with ARS: dose range of 0.1–7.1 Gy (mean ± SD: 2.0 ± 1.9 Gy).	5
**Loganovsky et al., 2000** [72]	Ukraine	Cross-sectional study	100 patients with ARS and 100 liquidators compared with a control group (*n* = 20) and with veterans of the Afghanistan war with PTSD (*n* = 50) and veterans with both PTSD and closed head injury (*n* = 50)	100 patients with ARS: absorbed doses up to 6.6 Gy 54 of the 100 liquidators were chronically irradiated at doses below 0.30 Sv (average dose: 0.16 ± 0.05 Sv) and the 46 left were chronically irradiated above 0.30 Sv (average dose: 0.69 ± 0.15 Sv)	4
***Medical patients***					
**Tran et al., 2017** [73]	Canada and USA	Pooled cohort	28,229 (F), 30,447 (M) patients	Mean cumulative lung dose: 0.18 Gy (range: 0.01–0.50)	7

Abbreviations: M: male; F: Female; Gy: Gray; Sv: Sievert; NA: Not Available; EGG: electroencephalogram; ARS: acute radiation sickness; PTSD: post-traumatic stress disorders; SD: standard deviation; se: standard error; max: maximum; WLM: Working Level Month.

**Table 2:** Key findings of the included studies on diseases of the nervous system.

First Author, Year	Outcomes(s)	Major Results	Confounding Factors Included in ERR Models
***Nuclear workers and uranium miners***			
**Boice, Cohen, Mumma, Hagemeyer et al., 2021** [32]	1—Dementia and Alzheimer’s disease (ICD-9: 290.0–290.4, 331.0) 2—Dementia, Alzheimer’s, Parkinson’s, and other motor neuron diseases (ICD-9: 290.0–290.4, 331.0, 332, 335.2) 3—Diseases of the nervous system and sense organs (ICD-9: 320–389) 4—Parkinson’s disease (ICD-9: 332)	1—SMR (95% CI): 0.92 (0.84, 1.02), n_deaths_ = 411 2—SMR (95% CI): 0.93 (0.86, 1.00), n_deaths_ = 657 3—SMR (95% CI): 0.82 (0.76, 0.89), n_deaths_ = 673 4—SMR (95% CI): 0.90 (0.76, 1.06), n_deaths_ = 140; HR (95% CI) at 100 mGy: 1.27 (0.98, 1.65); ERR (95% CI) at 100 mGy: 0.24 (−0.02, 0.50)	Sex, year of birth, SES, with a 10-year lag
**Boice, Cohen, Mumma, Golden et al., 2021** [33]	1—Dementia and Alzheimer’s disease (ICD-9: 290.0–290.4, 331.0) 2—Dementia, Alzheimer’s, Parkinson’s, and other motor neuron diseases (ICD-9: 290.0–290.4, 331.0, 332, 335.2) 3—Diseases of the nervous system and sense organs (ICD-9: 320–389) 4—Parkinson’s disease (ICD-9: 332)	1—SMR (95% CI): 0.92 (0.85, 0.98), n_deaths_ = 735 2—SMR (95% CI): 0.96 (0.90, 1.02), n_deaths_ = 973; HR (95% CI) at 100 mGy: 0.99 (0.83, 1.18); ERR (95% CI) at 100 mGy: −0.01 (−0.19, 0.16) 3—SMR (95% CI): 0.97 (0.90, 1.03), n_deaths_ = 815 4—SMR (95% CI): 1.16 (1.00, 1.34), n_deaths_ = 193; HR (95% CI) at 100 mGy: 1.18 (0.93, 1.49); ERR (95% CI) at 100 mGy: 0.16 (−0.07, 0.40)	Sex, year of birth, education, with a 10-year lag
**Kreuzer et al., 2021** [34]	1—Diseases of the nervous system (ICD-10: G00–G99) 2—Amyotrophic lateral sclerosis (ICD-10: G12.2)	1—SMR (95% CI): 0.73 (0.62, 0.85), n_deaths_ = 163 2—SMR (95% CI): 1.10 (0.67, 1.70) n_deaths_ = 20	
**Azizova et al., 2020** [12]	Incidence from Parkinson’s disease (ICD-10: G20)	ERR (95% CI) per Gy = 1.03 (95% CI: 0.60, 1.64), n_diseases_ = 300	Sex, attained age, with a 10-year lag
**Kelly-Reif et al., 2019** [35]	Diseases of the nervous system and sense organs (ICD-9: 320–389)	SMR (95% CI): 0.72 (0.39, 1.04), n_deaths_ = 19	
**Golden et al., 2019** [36]	1—Disease of the nervous system and sense organs (ICD-9: 320–389) 2—Dementia and Alzheimer’s disease (ICD-9: 290, 331) 3—Dementia, Alzheimer’s, Parkinson’s and motor neuron diseases (290, 331, 332, 335.2)	1—SMR (95% CI): 1.14 (0.89, 1.43), n_deaths_ = 72 2—SMR (95% CI): 1.18 (0.88, 1.55), n_deaths_ = 50 3—SMR (95% CI): 1.17 (0.91, 1.48) n_deaths_ = 71; HR (95% CI) at 100 mGy: 0.91 (0.64, 1.29); ERR (95% CI) at 100 mGy: −0.13 (−0.28, 0.02)	Year of birth and pay-type (hourly vs. salary)
**Gillies et al., 2017** [39]	Mortality from disease of the nervous system and sense organs (ICD-9: 320–389/ICD-10: G00-H95)	ERR (90% CI) per Sv: −0.15 (<−0.68, 0.50), n_deaths_ = 1505	Age, birth-cohort, gender, socioeconomic status, duration of employment, facility of employment, with a 10-year lag
**Boice et al., 2014** [42]	Diseases of the nervous system and sense organs (ICD-9: 320–389)	SMR (95% CI) 0.99 (0.79, 1.24), n_deaths_ = 78	
**Boice et al., 2011** [44]	Diseases of the nervous system and sense organs (ICD-9: 320–389)	SMR (95% CI): 0.95 (0.74, 1.20), n_deaths_ = 71	
**Lane et al., 2010** [45]	All nervous system diseases (ICD-NA)	SMR (95% CI): 0.66 (0.51, 0.85), n_deaths_ = 61	
**Schubauer-Berigan et al., 2009** [46]	Nervous system disorders (ICD-NA)	Whites: SMR (95% CI): 1.02 (0.70, 1.44), n_deaths_ = 32 American Indians: SMR (95% CI): 1.32 (0.63, 2.43), n_deaths_ = 10	
**Boice et al., 2008** [47]	Diseases of the nervous system and sense organs (ICD-9: 320–389)	SMR (95% CI): 1.60 (1.01, 2.39) (M/F), n_deaths_ = 23; SMR (95% CI): 1.52 (0.94, 2.32) (M), n_deaths_ = 21; SMR (95% CI): 3.29 (0.40, 11.9), n_deaths_ = 2 (F)	
**Howe et al., 2004** [49]	Nervous system disease (ICD: NA)	SMR (95% CI): 0.50 (0.31, 0.77), n_deaths_ = 20 RR (95% CI): dose group: <1 mSv: 1.00 (ref); 1–49 mSv: 1.08 (0.33, 3.54); 100-mSv: 3.25 (0.52, 20.29) ERR (95% CI) per Sv: 46.8 (1.51, 242)	Sex, age, calendar year, ethnicity, SES, facility, duration of monitoring, with a 10-year lag
***Nuclear weapons test participants***			
**Boice et al., 2020** [53]	1—Diseases of nervous system and sense organs (ICD-9: 320–389) 2—Dementia and Alzheimer’s disease (ICD-9: 290.0–290.4, 331.0)	1—SMR (95% CI): 0.84 (0.81, 0.88), n_deaths_ = 1871 2—SMR (95% CI): 0.90 (0.86, 0.95), n_deaths_ = 1330	
**Gun et al., 2008** [54]	1—Nervous system disease (ICD: NA) 2—Motor neuron disease (ICD: NA)	1—SMR (95% CI): 1.02 (0.78, 1.32), n_deaths_ = 59 2—SMR (95% CI): 1.24 (0.71, 2.02), n_deaths_ = 16	
***Medical workers***			
**Boice, Cohen, Mumma, Howard et al., 2021** [58]	1—Diseases of the nervous system and sense organs (ICD-9: 320–389) 2—Parkinson’s disease (ICD-9: 332) 3—Dementia and Alzheimer’s disease (ICD-9: 290.0–290.4, 331.0) 4—Dementia, Alzheimer’s, Parkinson’s, and other motor neuron diseases (ICD-9: 290.0–290.4, 331.0, 332, 335.2)	1—SMR (95% CI): 0.72 (0.65, 0.78), n_deaths_ = 471 2—SMR (95% CI): 0.82 (0.66, 1.02), n_deaths_ = 84; HR (95% CI) at 100 mGy: 1.18 (0.82, 1.71); ERR (95% CI) at 100 mGy: 0.17 (−0.20, 0.54) 3—SMR (95% CI): 0.70 (0.63, 0.79), n_deaths_ = 326 4—SMR (95% CI): 0.74 (0.68, 0.81), n_deaths_ = 476; HR (95% CI) at 100 mGy: 1.05 (0.88, 1.25); ERR (95% CI): 0.05 (−0.13, 0.23)	Sex, year of birth, occupational category, with a 10-year lag
**Lee et al., 2018** [60]	Diseases of the nervous system (ICD-10: G00–G99)	SMR (95% CI): 0.54 (0.33, 0.88), n_deaths_ = 16 (M)	
**Linet et al., 2017** [61]	Neurological and mental conditions (ICD: NA)	RR (95% CI): 0.94 (0.77, 1.15)	
**Berrington de González et al., 2016** [62]	1—Diseases of the nervous system (ICD-9: 320–389/ICD-10: G00–G99) 2—Other degenerative diseases of the nervous system (ICD-9: 331.1–337.9/ICD-10: G31–G32) 3—Alzheimer disease (ICD-9: 331.0/ICD-10: G30)	1—RR (95% CI): 1.04 (0.86, 1.25) 1—SMR (95% CI): 0.91 (0.78, 1.04) (M/F), n_deaths_ = 185 2—RR (95% CI): 1.39 (0.96, 2.01) 2—SMR (95% CI): 1.50 (1.09, 1.90) (M/F), n_deaths_ = 53 3—RR (95% CI): 0.94 (0.67, 1.33) 3—SMR (95% CI): 0.91 (0.66, 1.16) (M/F), n_deaths_ = 51	
**Wang et al., 2009** [64]	Diseases of the nervous system and sense organs (ICD-9: 320–386)	RR (95% CI): 2.10 (1.20, 3.10)	
***Chernobyl cleanup workers***			
**Rahu et al., 2014** [70]	1—Disease of the nervous system (ICD-10: G00–G99) 2—Degeneration of nervous system due to alcohol (ICD-10: G31.2) 3—Epilepsy (ICD-10: G40) 4—Migraine and other headache (ICD-10: G43–G44) 5—Nerve, nerve root and plexus disorders (ICD-10: G50–G59) 6—Sleep disorders (ICD-10: F51, G47)	1—RR (95% CI): 1.13 (1.06, 1.21) 2—RR (95% CI): 1.51 (1.04, 2.18) 3—RR (95% CI): 1.40 (1.14, 1.73) 4—RR (95% CI): 1.03 (0.83, 1.28) 5—RR (95% CI): 1.15 (1.02, 1.29) 6—RR (95% CI): 1.08 (0.93, 1.25)	

Abbreviations: M: male; F: Female; Gy: Gray; Sv: Sievert; ICD: International Classification of Diseases; RR: Relative Risk; SMR: Standardized Mortality Ratio; ERR: Excess Relative Risk; NA: Not Available; HR: Hazard ratios; SD: standard deviation; max: maximum; SES: socioeconomic status.

**Table 3:** Key findings of the included studies on cerebrovascular diseases.

First Author, Year	Outcomes(s)	Major Results	Confounding Factors Included in ERR Models
***Nuclear workers and uranium miners***			
**Azizova et al., 2022** [30]	Incidence from cerebrovascular diseases (ICD-10: I60–I69)	External exposure: ERR (95% CI) per Gy: 0.39 (0.31, 0.48) (M/F); 0.37 (0.27, 0.47) (M); 0.47 (0.31, 0.66) (F) Internal exposure: ERR (95% CI) per Gy: 0.32 (0.16, 0.51) (M/F); 0.31 (0.10, 0.59) (M); 0.32 (0.11, 0.61) (F)	Sex, attained age, calendar period, smoking status, alcohol consumption status, gamma/alpha doses, with a 10-year lag
**Hinksman et al., 2022** [31]	Mortality from cerebrovascular diseases (ICD-9: 430.0–438.9)	ERR (95% CI) per Sv: 0.57 (0.00, 1.31), n_deaths_= 3219; SMR (95% CI): 0.87 (0.84, 0.90), n_deaths_ = 3219	Calendar period, attained age, sex, employment length, first employer, industrial category, with a 10-year lag
**Boice, Cohen, Mumma, Hagemeyer et al., 2021** [32]	Cerebrovascular diseases (ICD-9: 430–438)	SMR (95% CI): 0.88 (0.83, 0.94), n_deaths_ = 1078	
**Boice, Cohen, Mumma, Golden et al., 2021** [33]	Cerebrovascular diseases (ICD-9: 430–438)	SMR (95% CI): 0.72 (0.68, 0.77), n_deaths_ = 871; HR (95% CI) at 100 mGy: 0.89 (0.71, 1.13); ERR (95% CI) at 100 mGy: −0.11 (−0.35, 0.12)	Sex, year of birth, education, with a 10-year lag
**Kreuzer et al., 2021** [34]	Cerebrovascular diseases (ICD-10: I60–I69)	SMR (95% CI): 1.33 (1.26, 1.41), n_deaths_ = 1335	
**Kelly-Reif et al., 2019** [35]	Cerebrovascular diseases (ICD-9: 430–438)	SMR (95% CI): 0.35 (0.29, 0.41), n_deaths_ = 148	
**Golden et al., 2019** [36]	Cerebrovascular diseases (ICD-9: 430–438)	SMR (95% CI): 1.03 (0.85, 1.24), n_deaths_ = 114	
**Bouet et al., 2018** [37]	Cerebrovascular diseases (ICD- 9: 430–438/ICD-10: I60–I69, G45 except G45.3 and G45.4, G46)	SMR (95% CI): 0.79 (0.52, 1.15), n_deaths_ = 27	
**Rage et al., 2017** [38]	Mortality from cerebrovascular diseases (ICD-10: I60–I69)	SMR (95% CI): 0.96 (0.78, 1.16), n_deaths_ = 105; ERR (95% CI) per 100 WLM: 0.42 (0.04, 1.04)	Unadjusted ERR model, with a 10-year lag.
**Gillies et al., 2017** [39]	Mortality from cerebrovascular diseases (ICD-9: 430–438/ICD-10: I60–I69)	ERR per Sv (90% CI): 0.49 (0.11, 0.92), n_deaths_ = 4399	Age, birth-cohort, gender, socioeconomic status, duration of employment, and facility of employment, with a 10-year lag
**Navaranjan et al., 2016** [40]	Mortality from cerebrovascular diseases (ICD-9: 430–438)	SMR (95% CI): 0.76 (0.68, 0.84), n_deaths_ = 315; ERR (95% CI) per 100 WLM: 0.22 (−0.12, 0.58)	calendar period, attained age, with a 10-year lag
**Azizova et al., 2014** [41]	Mortality from cerebrovascular diseases (ICD-9: 430–438)	RR (95% CI) by total whole body external gamma dose: <0.1 Gy: 1; 0.1–0.2 Gy: 0.89 (0.73, 1.09); 0.2–0.5 Gy: 0.89 (0.74, 1.07) ERR (95% CI) per Gy: 0.05 (−0.03, 0.16), n_deaths_ = 632 RR (95% CI) by total absorbed alpha-particle dose in liver: <0.01 Gy: 1; 0.01–0.025 Gy: 1.15 (0.76, 1.76); 0.025–0.1 Gy: 1.16 (0.78, 1.76); >0.1 Gy: 1.40 (0.93, 2.16) ERR per Gy (95% CI): 0.17 (NA, 0.56)	
**Boice et al., 2014** [42]	Cerebrovascular diseases (ICD-9: 430–438)	SMR (95% CI): 0.83 (0.69, 0.98), n_deaths_ = 131	
**Kreuzer et al., 2013** [43]	Mortality from cerebrovascular diseases (ICD-10: I60–I69)	ERR (95% CI) per Sv: 0.44 (−0.16, 1.04), n_deaths_ = 2073; ERR (95% CI) per 100 WLM: 0.000 (−0.008, 0.009); ERR (95% CI) per 100 kBqh/m^3^: 0.12 (−0.41, 0.65) Cumulative exposure to external gamma radiation in mSv: RR (95% CI): Ref: 1; >0–50: 1.08 (0.95, 1.21); 50–100: 1.12 (0.93, 1.31); 100–150: 1.02 (0.77, 1.27); 150–200: 0.90 (0.61, 1.18); 200–300: 1.41 (1.08, 1.74); 300–400: 0.87 (0.53, 1.21); 400–909: 1.35 (0.80, 1.90)	Age, calendar year, with a 10-year lag
**Boice et al., 2011** [44]	Cerebrovascular diseases (ICD-9: 430–438)	SMR (95% CI): 0.73 (0.60, 0.87), n_deaths_ = 119	
**Lane et al., 2010** [45]	Stroke (ICD-NA)	SMR (95% CI): 0.79 (0.69, 0.89), n_deaths_ = 244. Radon decay products: ERR per 100 WLM: −0.04. *p* value: 0.012; gamma-ray dose: ERR (95% CI) per Sv: −0.29 (<−0.29, 0.27), *p* value: 0.21.	Sub-cohort, age at risk, calendar year at risk and duration of employment, with a 5-year lag (ERR/100 WLM) and a 2-year lag (ERR/Sv)
**Schubauer-Berigan et al., 2009** [46]	Cerebrovascular diseases (ICD-NA)	Whites: SMR (95% CI): 0.98 (0.78, 1.23), n_deaths_ = 78 American Indians: SMR (95% CI): 0.53 (0.29, 0.90), n_deaths_ = 14	
**Boice et al., 2008** [47]	Cerebrovascular diseases (ICD-9: 430–438)	SMR (95% CI): 0.98 (0.67, 1.38), n_deaths_ = 32 (M/F); SMR (95% CI): 0.95 (0.64, 1.36), n_deaths_ = 30 (M); SMR (95% CI): 1.61 (0.20, 5.81), n_deaths_ = 2 (F)	
**Villeneuve et al., 2007** [48]	Mortality from cerebrovascular diseases (ICD-9: 430–438)	Cumulative exposure to radon (in WLM). RR (95% CI): 0: 1.00 (ref); >0–100: 0.63 (0.30, 1.32); >100–400: 0.73 (0.32, 1.66); >800–1600: 0.49 (0.18, 1.34)	
**Howe et al., 2004** [49]	All vascular lesions of CNS (ICD: NA)	SMR (95% CI): 0.27 (0.17, 0.40), n_deaths_ = 24; ERR (95% CI) per Sv: −2.05 (<−2.06, 353); RR (95% CI): dose group: <1 mSv: 1.00 (ref); 1–49 mSv: 1.89 (0.52, 6.83); 100—mSv: 3.27 (0.48, 22.26)	Sex, age, calendar year, ethnicity, SES, facility, duration of monitoring, with a 10-year lag
***Atomic bomb survivors***			
**Shimizu et al., 2010** [51]	1—Mortality from cerebrovascular diseases (ICD-9: 430–438) as underlying cause of death 2—Mortality from cerebrovascular diseases (ICD-9: 430–438) as underlying or contributing cause of death	1—ERR (95% CI) per Gy: 0.09 (0.01, 0.17), n_deaths_ = 9622 2—ERR (95% CI) per Gy: 0.12 (0.05, 0.19), n_deaths_ = 12,139	City, sex, age at exposure and attained age
***Nuclear weapons test participants***			
**Gillies et al., 2022** [52]	Cerebrovascular diseases (ICD: NA)	SMR (95% CI): 0.91 (0.85, 0.97), n_deaths_ = 816 RR (90% CI): 1.12 (1.03, 1.21)	
**Boice et al., 2020** [53]	Cerebrovascular diseases (ICD-9: 430–438)	SMR (95% CI): 0.86 (0.83, 0.89), n_deaths_ = 3161	
**Gun et al., 2008** [52]	Cerebrovascular disease (ICD: NA)	SMR (95% CI): 0.86 (0.76, 0.98), n_deaths_ = 243	
***Flight attendants***			
**Hammer et al., 2014** [55]	Cerebrovascular diseases (ICD-9: 430–438/ICD-10: I60–I69)	Male cockpit: SMR_c_ (95% CI): 0.50 (0.41, 0.62), n_deaths_ = 132 Male cabin crew: SMR_c_ (95% CI): 0.77 (0.53, 1.09), n_deaths_ = 45 Female cabin crew: SMR_c_ (95% CI): 0.47 (0.33, 0.67), n_deaths_ = 45	
**Yong et al., 2014** [56]	Cerebrovascular diseases (ICD: NA)	SMR (95% CI): 0.61 (0.50, 0.74), n_deaths_ = 108	
***Environmental exposure***			
**Kim et al., 2020** [57]	Morbidity from stroke (ICD: NA)	OR= 1.004; 95% CI: 1.001, 1.007	
***Medical workers***			
**Boice et al., 2021** [58]	Cerebrovascular diseases (ICD-9: 430–438)	SMR (95% CI): 0.62 (0.57, 0.68), n_deaths_ = 462; HR (95% CI) at 100 mGy: 1.04 (0.86, 1.26); ERR (95% CI) at 100 mGy: 0.04 (−0.16, 0.23)	Sex, year of birth and occupational category, with a 10-year lag
**Cha et al., 2020** [59]	Morbidity from cerebrovascular diseases (ICD10: I60–I69)	ERR (95% CI) per 100 mGy: 3.10 (−0.75, 11.59), n_deaths_ = 109 (M/F); ERR (05% CI) per 100 mGy: 3.72 (−0.59, 14.15), n_deaths_ = 87 (M); ERR (95% CI) per 100 mGy: −2.99 (<−3.57, 25.52), n_deaths_ = 22; RR (95% CI): 1.11 (0.68, 1.81) (M)	Attained age, sex, and birth year, with a 10-year lag
**Lee et al., 2018** [60]	Cerebrovascular diseases (ICD-10: I60–I69)	SMR (95% CI): 0.37 (0.29, 0.49), n_deaths_ = 55 (M)	
**Linet et al., 2017** [61]	Cerebrovascular diseases (ICD: NA)	RR (95% CI): 0.91 (0.75, 1.09) SMR (95% CI): 0.42 (0.36, 0.49), n_deaths_ = 173	
**Berrington de González et al., 2016** [62]	Cerebrovascular diseases (ICD-9: 430–438/ICD-10: I60–I69)	RR (95% CI): 1.12 (0.95, 1.32) (M); RR (95% CI): 0.28 (0.08, 0.92) (F); SMR (95% CI): 0.52 (0.45, 0.59), n_deaths_ = 242 (M/F)	
**Rajaraman et al., 2016** [63]	Stroke (ICD-9: 430–434, 436/ICD-10: I60–I64)	Incidence: HR (95% CI): 1.34 (1.10, 1.64) Mortality: HR (95% CI): 1.22 (0.85, 1.73)	
**Wang et al., 2009** [64]	Cerebrovascular diseases (ICD-9: 430–438)	RR (95% CI): 1.40 (1.20, 1.50)	
***Chernobyl cleanup workers***			
**Loganovsky et al., 2020** [65]	1—Acute cerebrovascular disorders (ICD-9: 430.0–436.9/ICD-10: I60.0–I66.0) 2—Chronic cerebrovascular disorders and sequelae of cerebrovascular disease (ICD-9: 438.0–439.9/ICD-10: I67, I69)	1—RR (95% CI): 1.40 (1.30, 1.50) 2—RR (95% CI): 1.23 (1.00, 1.50)	
**Buzunov et al., 2018** [66]	Morbidity from cerebrovascular diseases (ICD: NA)	Age at the time of the accident: 18–60 years. Thyroid radiation dose: 0.31–0.75 Gy: RR (95% CI): 2.16 (1.45, 3.22); 0.76–2.00 Gy: RR (95% CI): 0.63 (0.39, 1.02)	
**Kashcheev et al., 2016** [67]	Incidence from cerebrovascular diseases (ICD-10: I60–I69)	ERR (95% CI) per Gy: 0.45 (0.28, 0.62), n_diseases_ = 23,264	Unadjusted ERR model
**Rahu et al., 2014** [70]	Cerebrovascular diseases (ICD-10: I60–I69)	RR (95% CI): 1.05 (0.91, 1.20)	
***Medical patients***			
**Tran et al., 2017** [73]	Mortality from cerebrovascular diseases (ICD-9: 430–438)	ERR (95% CI) per Gy, dose range (in Gy): 0–0.10: 1.998 (−2.102, 7.027); 0–0.20: 0.979 (−1.043, 3.453); 0–0.30: 0.915 (−0.101, 2.109); 0–0.40: 0.661 (−0.059, 1.499); 0–0.50: 0.441 (−0.119, 1.090)	Cohort/sub-cohort, gender, smoking status, tuberculosis status, attained age, calendar year at risk

Abbreviations: M: male; F: Female; Gy: Gray; Sv: Sievert; ICD: International Classification of Diseases; RR: Relative Risk; SMR: Standardized Mortality Ratio; ERR: Excess Relative Risk; NA: Not Available; SMR_C_: SMR corrected; HR: Hazard ratios; OR: Odds ratio; SD: Standard deviation; max: maximum; SES: socioeconomic status.

**Table 4:** Key findings of the included studies on mental and behavioral disorders.

First Author, Year	Outcomes(s)	Major Results	Confounding Factors Included in ERR Models
***Nuclear workers and uranium miners***			
**Boice, Cohen, Mumma, Hagemeyer et al., 2021** [32]	1—Mental and behavioral disorders (ICD-9: 290–319) 2—Dementia and Alzheimer’s diseases (ICD-9: 290.0–290.4, 331.0) 3—Dementia, Alzheimer’s, Parkinson’s, and other motor neuron diseases (ICD-9: 290.0–290.4, 331.0, 332, 335.2)	1—SMR (95% CI): 0.77 (0.70, 0.85), n_deaths_ = 425 2—SMR (95% CI): 0.92 (0.84, 1.02), n_deaths_ = 411 3—SMR (95% CI): 0.93 (0.86, 1.00), n_deaths_ = 657	
**Boice, Cohen, Mumma, Golden et al., 2021** [33]	1—Mental and behavioral disorders (ICD-9: 290–319) 2—Dementia and Alzheimer’s diseases (ICD-9: 290.0–290.4, 331.0) 3—Dementia, Alzheimer’s, Parkinson’s, and other motor neuron diseases (ICD-9: 290.0–290.4, 331.0, 332, 335.2)	1—SMR (95% CI): 0.99 (0.91, 1.12), n_deaths_ = 520 2—SMR (95% CI): 0.92 (0.85, 0.98), n_deaths_ = 735 3—SMR (95% CI): 0.96 (0.90, 1.02), n_deaths_ = 973; HR (95% CI) at 100 mGy: 0.99 (0.83, 1.18); ERR (95% CI) at 100 mGy: −0.01 (−0.19, 0.16)	Sex, year of birth, education, with a 10-year lag
**Kreuzer et al., 2021** [34]	Mental and behavioral disorders (ICD-10: F00–F99)	SMR (95% CI): 0.81 (0.70, 0.94), n_deaths_ = 191	
**Kelly-Reif et al., 2019** [35]	1—Mental and behavioral disorders (ICD-9: 290–319) 2—Alcohol dependence syndrome (ICD-9: 303)	1—SMR (95% CI): 1.88 (1.05, 2.71), n_deaths_ = 20 2—SMR (95% CI): 0.77 (0.20, 1.34), n_deaths_ =7	
**Golden et al., 2019** [36]	1—Mental and behavioral disorders (ICD-9: 290–319) 2—Dementia and Alzheimer’s diseases (ICD-9: 290, 331) 3—Dementia, Alzheimer’s, Parkinson’s, and other motor neuron diseases (290, 331, 332, 335.2)	1—SMR (95% CI): 1.04 (0.72, 1.47), n_deaths_ = 33 2—SMR (95% CI): 1.18 (0.88, 1.55), n_deaths_ = 50 3—SMR (95% CI): 1.17 (0.91, 1.48), n_deaths_= 71; HR (95% CI) at 100 mGy: 0.91 (0.64, 1.29); ERR (95% CI) at 100 mGy: −0.13 (−0.28, 0.02)	Year of birth, pay-type (hourly vs. salary)
**Gillies et al., 2017** [39]	Mortality from mental and behavioral disorders (ICD-9: 290–319/ICD-10: F00–F99)	ERR (90% CI) per Sv: 1.30 (0.23, 2.72), n_deaths_ = 705	Age, birth-cohort, gender, socioeconomic status, duration of employment, facility of employment, with a 10-year lag
**Boice et al., 2014** [42]	Mental and behavioral disorders (ICD-9: 290–319)	SMR (95% CI): 0.88 (0.61, 1.24), n_deaths_ = 33	
**Boice et al., 2011** [44]	Mental and behavioral disorders (ICD-9: 290–319)	SMR (95% CI): 1.28 (0.93, 1.72), n_deaths_ = 43	
**Lane et al., 2010** [45]	All mental disorders (ICD-NA)	SMR (95% CI): 0.44 (0.29, 0.63), n_deaths_ = 29	
**Schubauer-Berigan et al., 2009** [46]	Other mental disorders (ICD-NA)	Whites: SMR (95% CI): 1.54 (0.90, 2.47), n_deaths_ = 17 American Indians: SMR (95% CI): 0.00 (0.00, 0.94), n_deaths_ = 0	
**Boice et al., 2008** [47]	Mental and behavioral disorders (ICD-9: 290–319)	SMR (95% CI): 1.10 (0.50, 2.08), n_deaths_ = 9 (M/F); SMR (95% CI): 1.13 (0.52, 2.14) (M), n_deaths_ = 9; SMR (95% CI): 0.00 (NA, NA), n_deaths_ = 0 (F)	
**Sibley et al., 2003** [50]	Mortality from dementia (ICD-9: 290.0–290.1)	Max annual (OR = 2.11 (95% CI: 0.98, 4.40)) and total lifetime radiation doses (OR = 2.09 (95% CI: 1.02, 4.29))	
***Nuclear weapons test participants***			
**Boice et al., 2020** [53]	1—Mental and behavioral disorders (ICD-9: 290–319) 2—Dementia and Alzheimer’s diseases (ICD-9: 290.0–290.4, 331.0)	1—SMR (95% CI): 0.92 (0.87, 0.98), n_deaths_ = 1021 2—SMR (95% CI): 0.90 (0.86, 0.95), n_deaths_ = 1330	
***Medical workers***			
**Boice et al., 2021** [58]	1—Mental and behavioral disorders (ICD-9: 290–319) 2—Dementia and Alzheimer’s diseases (ICD-9: 290.0–290.4, 331.0) 3—Dementia, Alzheimer’s, Parkinson’s, and other motor neuron diseases (ICD-9: 290.0–290.4, 331.0, 332, 335.2)	1—SMR (95% CI): 0.58 (0.51, 0.66), n_deaths_ = 246 2—SMR (95% CI): 0.70 (0.63, 0.79), n_deaths_ = 326 3—SMR (95% CI): 0.74 (0.68, 0.81), n_deaths_ = 476; HR (95% CI) at 100 mGy: 1.05 (0.88, 1.25); ERR (95% CI) at 100 mGy: 0.05 (−0.13, 0.23)	
**Linet et al., 2017** [61]	Mental and behavioral disorders (ICD: NA)	RR (95% CI): 0.55 (0.35, 0.84)	
**Berrington de González et al., 2016** [62]	Mental and behavioral disorders (ICD-9: 290–319/ICD-10: F00–F99)	RR (95% CI): 1.30 (0.60, 2.80) SMR (95% CI): 0.51 (0.38, 0.64), n_deaths_ = 60	
***Chernobyl cleanup workers***			
**Loganovsky et al., 2020** [65]	1—Psychosis (ICD-9: 293.0–294.9/ICD-10: F00–F05, F06.0, F06.2) 2—Non-psychotic disorders (ICD-9: 310.0–310.9/ICD-10: F06.32, F06.4-F06.7, F07.7, F07.8, F07.9)	1—RR (95% CI): 3.15 (2.60, 3.70) 2—RR (95% CI): 1.99 (1.60, 2.50)	
**Loganovsky et al., 2016** [68]	Mild cognitive deficits (ICD-10: F06.7) Tests: Rey Auditory Verbal Learning Test (RAVLT), General Health Questionnaires (GHQ-28), Zung Self-Rating Depression Scale (SDS), Brief Psychiatric Rating Scale (BPRS)	Neurocognitive tests revealed the presence of mild cognitive disorders at t1: t0: 3.6%; t1: 11.2%, X^2^: 8.38, *p* < 0.01 and significant decrease in verbal learning: memorized words (mean ± SD): 10.43 ± 2.09 and 9.86 ± 1.70 at t0 and t1, respectively.	
**Bazyka et al., 2015** [69]	Neurocognitive and psychological tests: General Health Questionnaire (GHQ-28), Brief Psychiatric Rating Scale (BPRS-18), Rey Auditory Verbal Learning Test (RAVLT), Zung scores, Mini-mental State Examination (MMSE)	Cognitive functions in cleanup workers are characterized by symptoms of a mild cognitive impairment and a significantly higher level of mental disorders	
**Rahu et al., 2014** [70]	1 - Mental disorders (ICD-10: F00–F99) 2 - Mental disorders due to alcohol (F10) 3 - Depressive disorders (F32-F33) 4 - Anxiety disorders (F41) 5 - Stress reactions (F43)	1 - RR (95% CI): 1.00 (0.94, 1.07) 2 - RR (95% CI): 1.21 (1.06, 1.39) 3 - RR (95% CI): 0.97 (0.84, 1.11) 4 - RR (95% CI): 0.91 (0.74, 1.13) 5 - RR (95% CI): 0.72 (0.53, 0.97)	
**Loganovsky et al., 2013** [71]	Psychometric examinations were assessed by: Bried Psychiatric Rating Scale (BRPS), General Health Questionnaire (GHQ-28), Zung Self-Rating Depression Scale (SDS), Impact of Events Scale (IES and IES-R), PTSD scales Irritability, Spilberger-Khainin anxiety scale, Neurometric examination was performed using the neurological by: Functional System Scale (FSS), Expanded Disability Status Scale (EDSS). Cognitive functions were assessed by the Rey Auditory Verbal Learning Test (RAVLT) and the Short Cognitive Performance Test (SKT). Neurophysiologic studies included 16-channel quantitative electroencephalography (qEEG) with brain mapping of the main frequency ranges spectral analysis.	PTSD following radiation emergency is characterized by comorbidity of psychopathology and neurocognitive deficit.	
**Loganovsky et al., 2000** [72]	Schizophrenia (ICD-9: 295/ICD-10: F20)	Relative risk of schizophrenia in liquidators greater than in the general population (2.4 for 1986–1997) and 3.4 for 1990–1997); 72% of liquidators had EEG abnormalities.	

Abbreviations: M: male; F: Female; Gy: Gray; Sv: Sievert; ICD: International Classification of Diseases; RR: Relative Risk; SMR: Standardized Mortality Ratio; ERR: Excess Relative Risk; NA: Not Available; HR: Hazard ratios; OR: Odds Ratio; SD: standard deviation; max: maximum; PTSD: Post Traumatic Stress Disorder.

## Data Availability

Not applicable.

## Supplementary Materials

The following supporting information can be downloaded at https://www.mdpi.com/article/10.3390/brainsci12080984/s1, Table S1: Preferred Reporting Items for Systematic Reviews and Meta-analyses (PRISMA) 2020 checklist; Table S2: List of causes of deaths studied and corresponding codes according to the International Classification of Diseases (ICD). Ref. [95] is cited in Supplementary Materials.
